# Structural Persistence Masks Commit-Point Chemical Transformation in Copper–Imidazolate Nanosheet Metal–Organic Frameworks

**DOI:** 10.1021/acsnano.6c06011

**Published:** 2026-06-23

**Authors:** Swaroop Chakraborty, Ana Guilherme Buzanich, Prathmesh Bhadane, Hiroto Kitaguchi, Sang Pham, Iuliia Mikulska

**Affiliations:** a School of Geography, Earth & Environmental Sciences, 1724 University of Birmingham, Edgbaston B15 2TT, U.K.; b 42220 Bundesanstalt für Materialforschung und -prüfung (BAM), Richard-Willstaetter-Straße 11, 12489 Berlin, Germany; c Materials Engineering, Indian Institute of Technology, Gandhinagar 382355, India; d Facility of Electron Microscopy, University of Birmingham, Edgbaston B15 2TT, U.K.; e Diamond Light Source, Harwell Science and Innovation Campus, Didcot OX11 0DE, U.K.

**Keywords:** metal−organic frameworks, transformation kinetics, X-ray absorption spectroscopy, rapid transformation, safe and sustainable by design

## Abstract

Metal–organic frameworks (MOFs) are often assessed for stability using end-point structural metrics, yet their exposure-relevant chemical identity at the metal center may evolve on much shorter time scales in realistic aqueous environments. Here we examine nanoscale copper–imidazolate (CuIm) MOFs transformation across three benchmark matrices representing an abiotic-to-biorelevant gradient: a freshwater-like groundwater matrix (borehole water), a marine-like high-salinity matrix (artificial seawater), and a ligand-rich, protein-free cell-culture medium (serum-free DMEM) used here as a chemically complex challenge matrix. Using *ex situ* time-resolved separation of particle-associated and dissolved/complexed fractions coupled with copper-centered speciation analysis, we resolve a distinct kinetic hierarchy. CuIm remains largely conserved in borehole water, exhibits gradual reorganization in artificial seawater, and undergoes rapid transformation in serum-free DMEM, with the particle-associated fraction converging to an apparent end-state spectrum by ∼4 h. Medium-dependent copper mobilization to the dissolved/complexed pool accompanies these speciation trajectories. End-point characterization further indicates that framework-like structural signatures can persist while surface chemistry is substantially altered, demonstrating a decoupling between long-range order and node/surface identity. Collectively, these findings show that CuIm follows matrix-selected transformation trajectories with pronounced early-time trajectory shifts and that stability assessments must be grounded in time-resolved chemical identity rather than end-point crystallinity alone.

## Graphical abstract

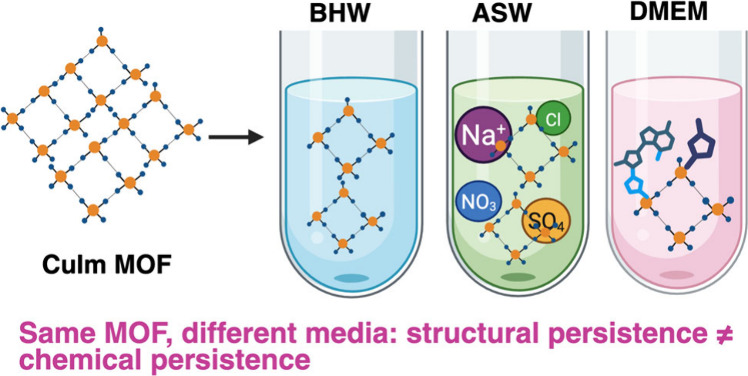

Metal–organic frameworks (MOFs), including nanoscale MOFs, are increasingly considered for applications that span environmental waters and biorelevant settings, from separations and sensing to adsorption-based remediation and biomedical concepts.^1,2^ Across these use cases, stability is often treated as a prerequisite for reliable function and safe translation because MOFs are generally expected to retain the structural and chemical features that underpin their intended performance. In practice, this means preserving adsorption-relevant porosity and framework integrity in environmental applications, or maintaining controlled degradation, colloidal behavior, and acceptable biocompatibility in biomedical settings. However, these assumptions can be misleading when stability is inferred mainly from retained crystallinity, because a material may appear structurally persistent while its local metal coordination environment and chemical identity have already changed.^3–5^ As a result, a material can retain a familiar structure while still undergoing chemically meaningful changes at the metal nodes that dictate reactivity, dissolution, and biological identity. Reviews of MOF water and chemical stability repeatedly emphasize that stability cannot be generalized without specifying the medium and the property being measured.^6,7^

A further complication is time scale. Transformation for engineered nanomaterials shows that many influential processes begin immediately on transfer into a new environment (adsorption of ligands/biomolecules, redox at exposed sites, diffusion-limited ligand exchange, and early surface restructuring), and these early events can determine the pathways and kinetics of later aggregation, dissolution, and secondary phase formation.^8,9^ In a recent perspective on rapid transformation dynamics, it is argued that conventional end-point characterization often misses the decisive early window, forcing risk-relevant interpretation to rely on retrospective snapshots that cannot faithfully reconstruct transformation trajectories.^10^ This limitation is particularly acute for MOFs entering physiological buffers or complete culture media, where phosphate, bicarbonate, amino acids, and biomacromolecules provide strong complexants that can drive ligand exchange and node/surface restructuring.^11^ Classic examples include imidazolate frameworks such as ZIF-8, which can undergo pronounced chemical/structural evolution in phosphate-buffered systems, highlighting that stable labels established in simpler waters do not automatically translate to biorelevant matrices.^12^

Copper–imidazolate nanosheet MOFs, referred to here as CuIm, are a stringent test case for this multiscale, time-dependent stability problem. The CuIm studied here is a copper­(II)–2-methylimidazolate/copper–imidazolate coordination framework prepared from copper­(II) nitrate and 2-methylimidazole precursors, in which Cu centers are connected through imidazolate nitrogen atoms within a nanosheet-like framework. Copper centers are functionally valuable yet coordination-active, meaning that local Cu chemistry (coordination environment, ligand field, and short-range order) can plausibly evolve rapidly under realistic media conditions even if long-range order appears preserved. For applications and safety-by-design decisions, the key exposure-relevant question is therefore not simply whether crystallinity is retained, but whether the Cu center preserves its chemical identity across environmental and biorelevant aqueous matrices, and how rapidly any change occurs. We hypothesized that CuIm can maintain apparent structural persistence while undergoing rapid, medium-dependent chemical transformation at Cu centers, with the fastest and most pronounced changes occurring in complex biological media on minute-to-hour time scales compared to environmental media.

To resolve this hypothesis, we performed an *ex situ*, time-resolved transformation study across an environmental-to-biological gradient using three representative matrices: borehole water (BHW), artificial seawater (ASW), and Dulbecco’s modified Eagle medium (DMEM). We used X-ray absorption spectroscopy (XAS) to characterize the electronic structure and local environment of copper. At defined exposure times (spanning the early-time window through 72 h), we tracked changes in the Cu-center electronic/local structure and compared them against bulk structural readouts, enabling a direct test of whether structural persistence is decoupled from chemical persistence at the reactive metal nodes. This design allows medium-specific transformation kinetics to be mapped and compared within a single framework material, addressing the broader call for transformation studies that explicitly capture early-time dynamics rather than relying solely on end-point characterization.

## Results and Discussion

### Characterization of CuIm MOFs

The structural and physicochemical properties of CuIm were systematically analyzed to evaluate its framework integrity and textural characteristics. Scanning electron microscopy (SEM) and transmission electron microscopy (TEM) images indicate heterogeneous, partially agglomerated nanosheet-like CuIm particles, predominantly in the ∼100–200 nm size range, rather than a strictly uniform morphology (Figure S1a,d). PXRD confirms the crystalline structure of the CuIm, with distinct reflections at 2θ = 14.8°, 30°, 31.6°, 33.3°, and 47.8°, indicating a well-defined coordination framework. The observed peak broadening, however, suggests reduced crystallite domain size and the presence of microstructural disorder, which is consistent with the formation of a more open architecture observed in surface area and porosity analysis. Because of the small crystallite size and nanosheet morphology, single-crystal XRD was not feasible in this study; instead, framework identity was assessed using PXRD, FTIR, Raman spectroscopy and Cu K-edge XAS (Figure S1b). Textural properties were investigated using N_2_ adsorption–desorption analysis (Figure S1c), which reveals a specific surface area of 70 m^2^ g^–1^ and a pore volume of 0.88 cm^3^ g^–1^, confirming the development of a porous structure. FT-IR spectroscopy further verifies the coordination environment within the material (Figure S1f). Within the measured 600–4000 cm^–1^ window, FTIR (Figure S1f) confirms the imidazolate linker fingerprint of CuIm, including bands in the ∼1600–1450 cm^–1^ region and features at ∼1420, ∼1305, and ∼1150 cm^–1^ consistent with linker-associated vibrations.^13^ The presence of accessible porosity suggests enhanced diffusion pathways and increased exposure of active sites. The TGA curve indicates that the material remains thermally stable up to approximately 230 °C, beyond which mass loss occurs due to removal of unreacted 2-mIm, followed by decomposition of the organic linker and eventual residue formation (Figure S1e). A schematic representation of the two-dimensional CuIm nanosheet layer is provided in Figure S7, highlighting the key Cu–N coordination environment between Cu centers and imidazolate linkers. This schematic provides a structural reference for the Cu-centered XAS and linear combination fitting (LCF) analysis used below to track framework chemical transformation.

### *Ex Situ* Time-Resolved Transformation by X-ray Absorption Spectroscopy (XAS)

Cu K-edge XAS containing both X-ray absorption near edge structure (XANES) and extended X-ray absorption fine structure (EXAFS) regions was performed to obtain information on the electronic structure and local environment around Cu atoms in the CuIm MOF. To isolate particle-associated transformation kinetics from the mobilized Cu pool, CuIm suspensions were sampled over time and separated using 0.02 μm filtration, yielding a membrane-retained solid fraction and a <0.02 μm filtrate. The 0.02 μm cutoff was selected as a stringent operational boundary because it is substantially smaller than conventional 0.22 or 0.45 μm filtration and therefore minimizes carry-through of parent nanosheet particles and larger transformed/aggregated Cu-bearing solids into the filtrate, while still enabling rapid time-resolved sampling. For Cu K-edge XAS analysis of the solid fraction collected at different time points, the syringe-filter housings were dismantled, and the membranes were mounted on the beamline’s sample holders for measurements to minimize artifacts from the polymer casing. In parallel, the final powders recovered after 72 h exposure remaining in each beaker were recovered and analyzed to establish the terminal Cu coordination environment in each matrix. This design directly addresses a known limitation in MOF stability screening: long-range crystallinity and linker fingerprints can persist while metal-node chemistry evolves substantially depending on medium composition and time window.^6^ Throughout this manuscript, the membrane-retained material is referred to as the particle-associated fraction, whereas the filtrate is defined as the <0.02 μm dissolved/complexed/ultrasmall-colloidal Cu fraction. This terminology acknowledges that the filtrate may contain Cu ions, Cu–ligand complexes, polynuclear Cu species and any ultrasmall Cu-bearing colloids smaller than the nominal filter cutoff, rather than purely ionic dissolved Cu. Importantly, the term particle-associated fraction is operational and does not imply that all retained Cu-bearing environments correspond to discrete crystalline secondary phases. In the LCF analysis below, reference spectra such as Cu­(II) oxide, Cu­(II) hydroxide, Cu­(II) carbonate or Cu­(I) chloride are used as chemically relevant spectral analogues for local Cu coordination environments. Therefore, fitted contributions are interpreted as oxide-like, hydroxide-like, carbonate-associated or chloride-like Cu motifs within heterogeneous retained material, rather than as definitive evidence for bulk crystalline CuO, Cu­(OH)_2_ or other stand-alone phases. This distinction is essential because Cu K-edge XAS is sensitive to short-range Cu coordination and electronic structure, whereas PXRD primarily reports long-range crystalline order.

Across the borehole water (BHW) exposure time series, the Cu K-edge XANES spectra of the filter-retained samples remain highly similar to pristine CuIm at the dominant Cu-centered XAS level with exposure time and closely match both pristine CuIm and the BHW final powder (Figure 1a). Only minor variations in intensity at the absorption maximum are observed, while the overall edge position and near edge profile remain unchanged. The corresponding Fourier-transformed extended X-ray absorption fine structure (FT-EXAFS) magnitudes show the same set of coordination-shell features and overall shape across 1 to 72 h, with only small reductions in amplitude (Figure 1b). These minor spectral differences should be interpreted with caution because the pristine and end-point powders and the time-resolved filter-retained solids were analyzed in different specimen formats, although both were evaluated in transmission mode. Under these conditions, small variations in specimen homogeneity, packing density, thickness uniformity, and effective absorption path length between cellulose-diluted pellets and filter-retained deposits may influence the apparent EXAFS amplitude and subtle near-edge intensities. Given that the edge position, overall, XANES profile shape, and coordination-shell pattern remain otherwise unchanged, these small differences are not considered sufficient evidence for a distinct Cu coordination environment in BHW. Collectively, the BHW data set supports a strong and simple conclusion: BHW does not induce major Cu-centered changes in the electronic state or local coordination environment of Cu in CuIm over 72 h, and the end-point powder remains closely comparable to pristine CuIm in its dominant XAS signature, within the sensitivity of Cu K-edge XAS (Figure 1a,b). However, BHW should not be interpreted as a completely inert or unchanged condition. The XPS surface atomic composition shows a modest increase in surface Cu from 1.8 to 3.6 atom % and O from 6.0 to 9.0 atom % after 72 h BHW aging (Table S1), while LCF indicates a small decrease in the CuIm-like contribution with minor non-CuIm-like coordination motifs (Figure 3). These changes are substantially smaller than those observed in ASW and especially serum-free DMEM, but they indicate limited surface hydration/oxygenation, increased near-surface Cu accessibility, and low-level surface/defect heterogeneity under BHW exposure. Thus, the appropriate interpretation is not that BHW causes no change at all but that it preserves the dominant CuIm-like Cu coordination environment while inducing only minor surface-level conditioning.

**Figure 1:**
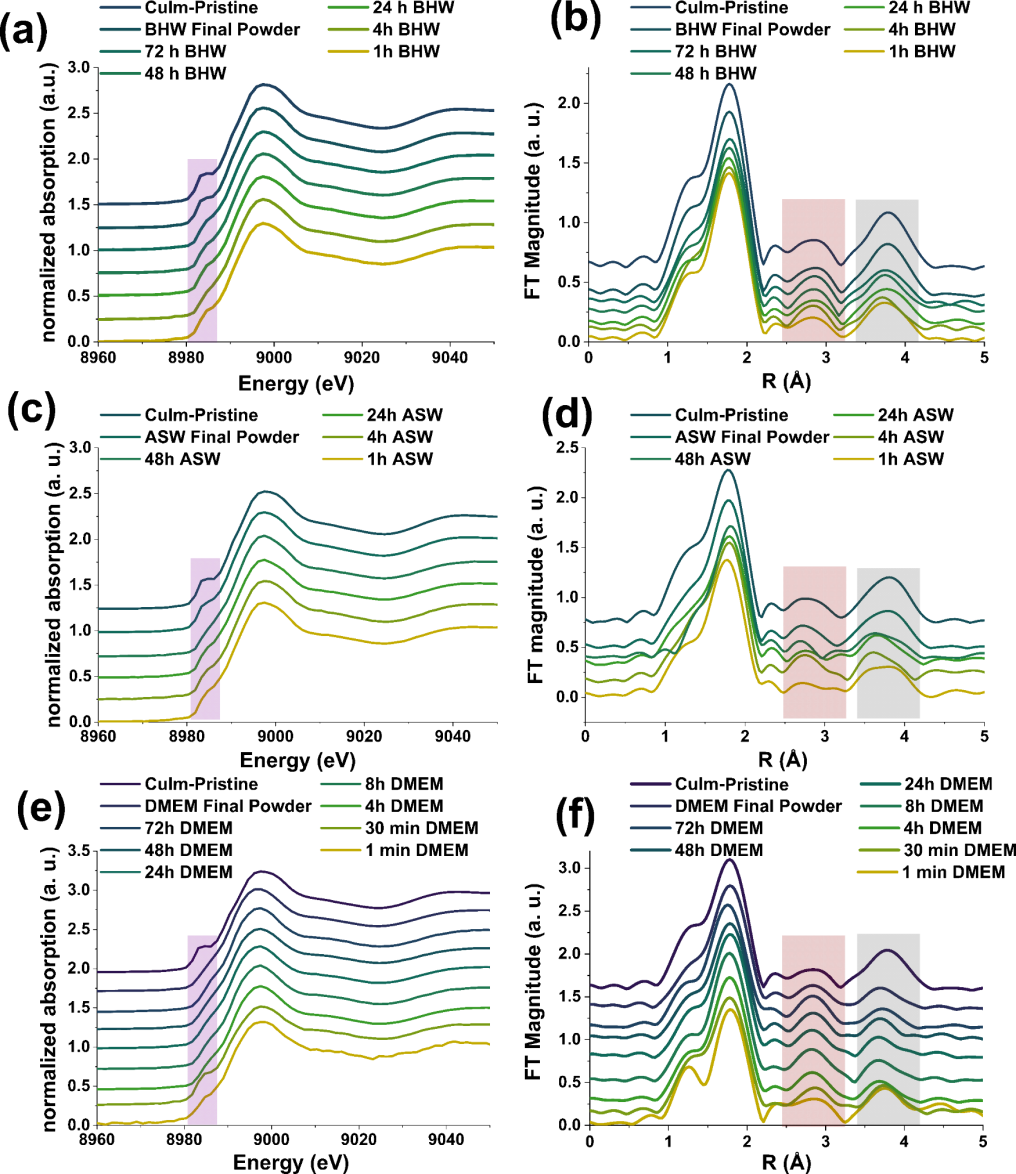
*Ex situ* time-resolved Cu K-edge XAS of filter-retained CuIm across different matrices. Cu K-edge (a, c, e) XANES spectra and (b, d, f) Fourier transform magnitude of *k* ^2^χ­(*k*)-weighted EXAFS spectra for the filter-retained (0.02 μm) solid fraction recovered from (a, b) borehole water (BHW), (c, d) artificial seawater (ASW), and (e, f) serum-free DMEM at indicated time points. Data are shown alongside the pristine CuIm and the 72 h end point (final powder) recovered from the exposure beaker. Spectra are vertically offset for clarity. The figure highlights a matrix-dependent kinetic hierarchy at the Cu center in CuIm MOF: dominant Cu-center conservation in BHW with only minor spectral/surface heterogeneity, slow/subtle second-shell reorganization in ASW, and rapid minute-to-hour restructuring in serum-free DMEM, where filter spectra converge to the final-powder signature by ∼4 h. The highlighted regions indicate the principal spectral features used to compare matrix-dependent changes relative to pristine CuIm.

**Figure 2:**
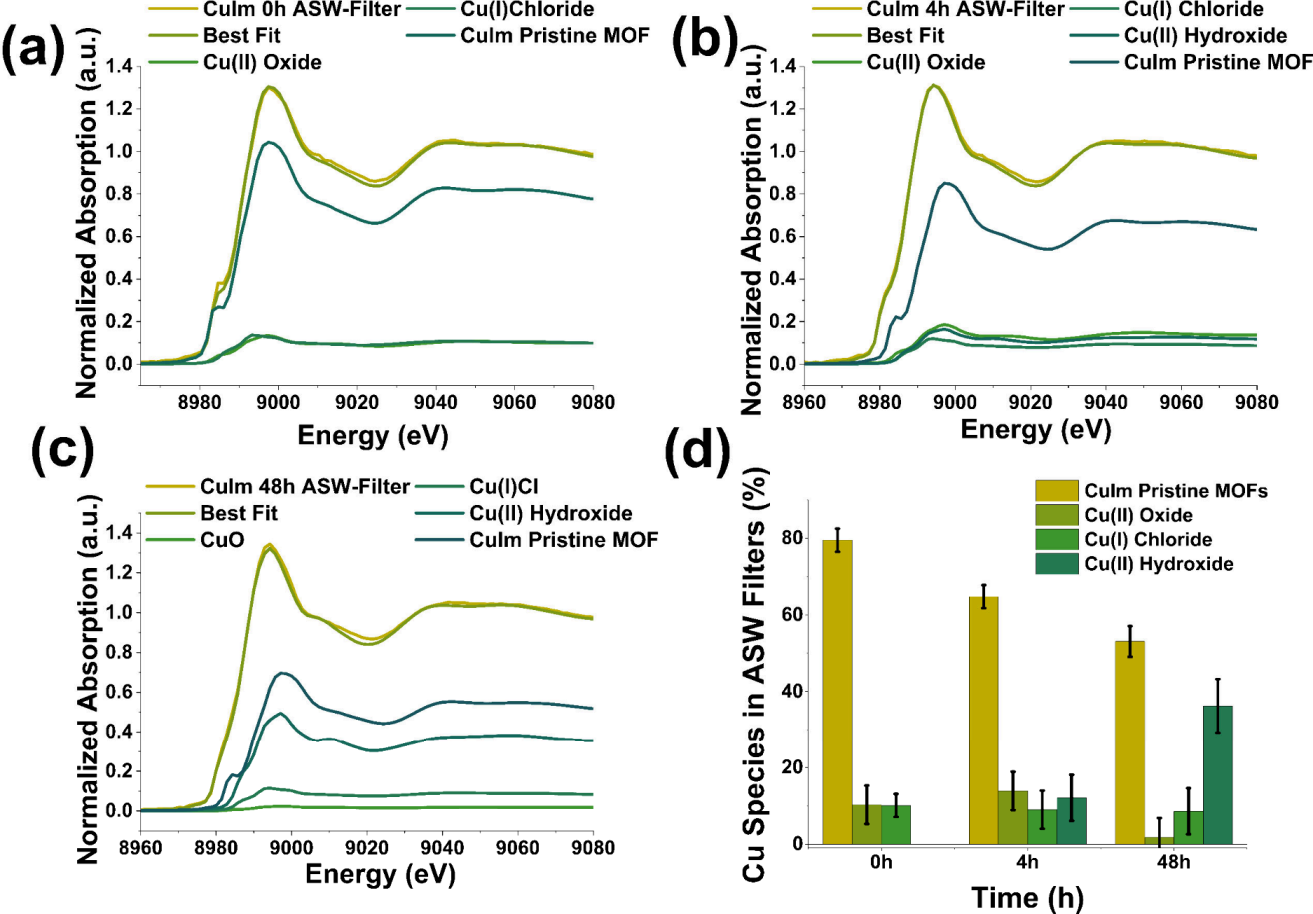
Cu K-edge XANES linear combination fitting (LCF) results for filter-retained solids collected throughout the aging of CuIm in ASW. (a–c) Measured spectra (black) and LCF best fit (red) for representative time points (0 h, 4 h, 48 h), shown with contributing reference spectra used in the fit (e.g., pristine CuIm, Cu­(I) chloride, Cu­(II) oxide, Cu­(II) hydroxide as indicated). (d) Summary of LCF-derived fractions (%) versus time, showing the progressive decrease in CuIm-like spectral contribution and concomitant increase of oxide-like/hydroxide-like or chloride-associated Cu coordination motifs under marine-like ionic strength conditions. These LCF components are interpreted as local coordination-environment analogues rather than definitive crystalline secondary phases. Corresponding LCF fit-quality parameters, including χ^2^ and reduced χ^2^ values for each fitted spectrum, are provided in Table S3.

**Figure 3:**
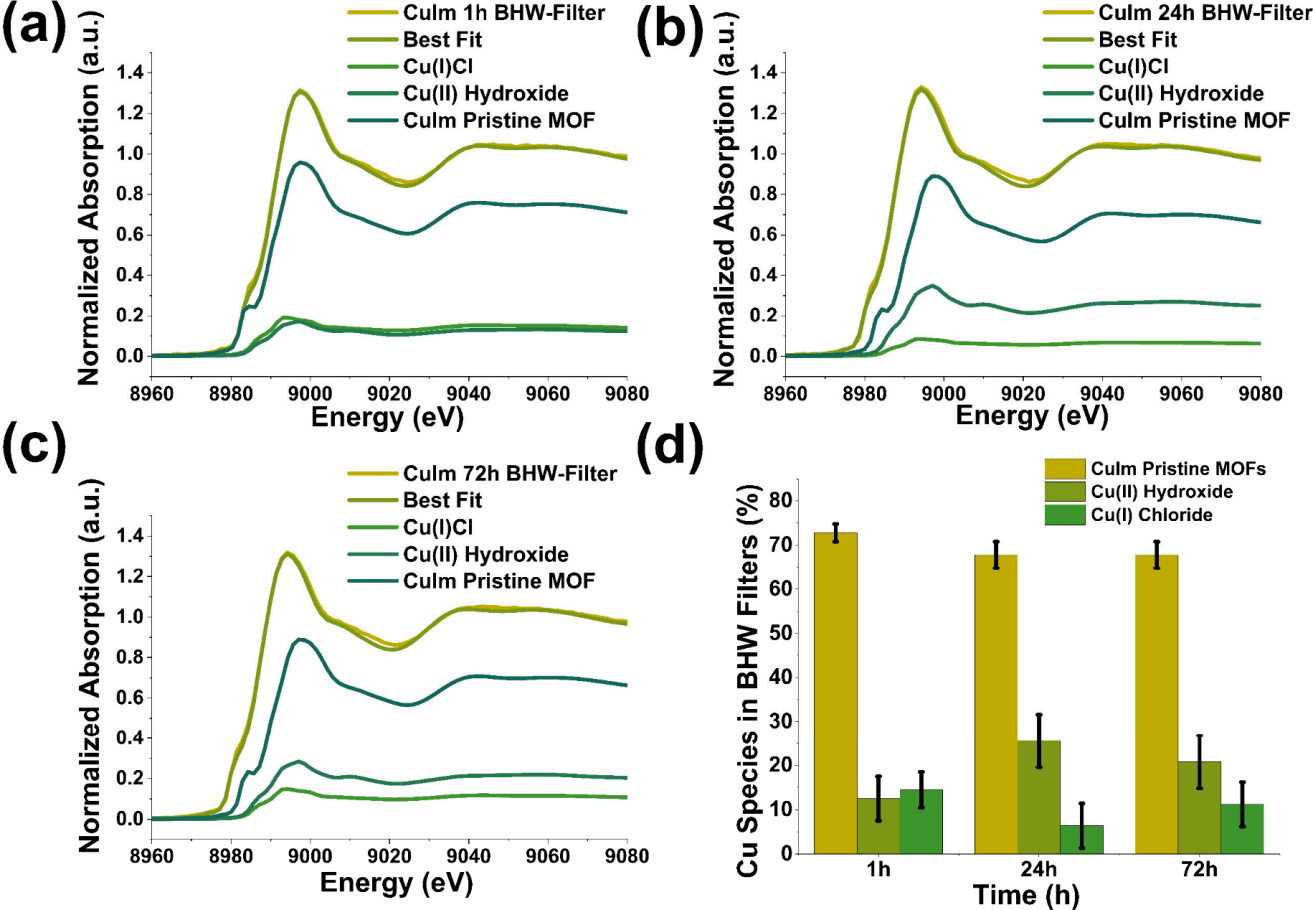
Linear combination fitting of particle-associated Cu speciation on filters during aging in BHW. Cu K-edge XANES LCF for filter-retained solids collected during CuIm aging in BHW. (a–c) Measured spectra (black) and LCF best fit (red) for representative time points (1 h, 24 h, 72 h), plotted with the reference components included in the fit (e.g., pristine CuIm, Cu­(I) chloride, Cu­(II) hydroxide as indicated). (d) LCF-derived fractions (%) versus time, showing that the particle-associated fraction remains predominantly CuIm-like throughout 72 h, with only minor contributions from non-CuIm-like spectral motifs, interpreted as small degrees of local coordination heterogeneity rather than formation of detectable bulk crystalline secondary phases. Corresponding LCF fit-quality parameters, including χ^2^ and reduced χ^2^ values for each fitted spectrum, are provided in Table S3.

In contrast, exposure to artificial seawater (ASW) results in consistent, time-dependent spectral evolution indicative of gradual changes in the local Cu coordination environment. The pristine and final powder after exposure to ASW show discernible differences in Cu K-edge XANES measurements, including a slight increase in white-line intensity and a marginal shift of the white line to lower energy relative to pristine CuIm (Figure 1c), consistent with a modified average electronic/coordination environment around Cu after prolonged contact with seawater ions. The clearest time-dependent signature is observed in the FT-EXAFS: the second-shell feature broadens and progressively redistributes from the pristine maximum near ∼3.8 Å toward a lower-R shoulder/component near ∼3.6 Å, while first-shell contributions are retained (Figure 1d). This evolution points to incipient local reorganization around Cu centers rather than loss of short-range order: first-shell contributions persist, while medium-range order becomes subtly redistributed, consistent with the onset of partial rearrangement or interaction with seawater-derived species. In other words, ASW appears to drive a slow trajectory in which CuIm remains largely preserved but accumulates measurable local structural changes over tens of hours.

The most pronounced and fastest transformation is observed in serum-free DMEM. The Cu K-edge XANES spectra show that changes initiate within 1 min of exposure: a low-energy shoulder in the near-edge region becomes visibly attenuated and continues to diminish with increasing contact time (Figure 1e). By 4 h, the XANES spectrum of the exposed material becomes indistinguishable from the DMEM final powder, indicating that the dominant transformation of the particle-associated Cu environment is essentially complete on an hour time scale and then persists with little further evolution up to 72 h (Figure 1e). The FT-EXAFS magnitudes corroborate this rapid restructuring. With increasing time in DMEM, the second coordination shell decreases in intensity and its peak position shows a small shift (from ∼3.8 Å to ∼3.7 Å) (Figure 1f). A coupled reduction and slight shift of higher-shell contributions is consistent with increasing disorder and/or a change in medium-range coordination motifs around Cu, as expected if ligand-rich components in DMEM promote partial restructuring or ligand exchange at coordination-active Cu sites. The key kinetic insight is the early convergence: by ∼4 h, the particle-associated spectrum becomes indistinguishable from the 72 h final powder, indicating that the dominant *ex situ* transformation trajectory has already been established by this time (Figure 1e,f). This type of buffer/media-driven redefinition without immediate collapse is well documented for imidazolate MOFs in phosphate-/bicarbonate-containing physiological environments and motivates time-resolved chemical (not just structural) stability assessment.^12,15^

Together, these data demonstrate that a distinct medium-dependent kinetic hierarchy was observed at the Cu centers. Bringing the filter-resolved time series together with the end-point powders reveals a coherent, matrix-controlled hierarchy in particle-associated Cu chemistry: dominant Cu-center conservation in BHW with minor surface/defect heterogeneity, slow and subtle evolution in ASW, and rapid minute-to-hour transformation in serum-free DMEM. This ordering is important because it shows that CuIm can exhibit apparent persistence in simpler waters while undergoing fast, ligand-driven reconfiguration in a physiological, chemically complex environment, even without serum proteins. Finally, to move from spectral evolution to chemically constrained assignments, we used LCF of Cu K-edge XANES against a reference library of plausible Cu end members (CuIm pristine plus representative Cu­(I)/Cu­(II) salts/oxides) for the liquid time series and selected solid time points, including the final powders. This provides the quantitative framework for tracking how the Cu speciation mix evolves with time and whether the particle-associated fraction remains CuIm-like or progressively incorporates secondary Cu environments building directly on the qualitative kinetic picture established here from XANES and EXAFS trends.^16,17^

### Linear Combination Fitting (LCF)

LCF was used here as a semiquantitative tool to describe the redistribution of Cu local coordination environments during aging, not as a crystallographic phase-quantification method. Because several Cu reference compounds can produce spectral fits of similar quality, the fitted components are interpreted conservatively as spectral analogues for local Cu environments. Thus, Cu­(II) oxide, Cu­(II) hydroxide, Cu­(II) carbonate, Cu­(II) phosphate, Cu­(II) nitrate, and Cu­(I) chloride references are discussed as oxide-like, hydroxide-like, carbonate-associated, phosphate-associated, nitrate-associated, and chloride-like Cu motifs, respectively, rather than as definitive stand-alone crystalline phases. The LCF results therefore indicate progressive reconfiguration of the average Cu coordination environment within the particle-associated fraction, which may include surface/edge restructuring, ligand exchange, defect-associated Cu–O­(H) environments, amorphous or poorly ordered secondary motifs, and retained CuIm-like domains. To support the reliability of the LCF-derived trends, fit-quality parameters for every LCF shown in Figures 2–4 and Figures S4 and S5 are summarized in Table S3. Across the full data set, reduced χ^2^ values remained in the range of 1.33 × 10^–5^ to 7.78 × 10^–4^, indicating consistent agreement between the experimental spectra and the fitted linear combinations. These statistics support the use of LCF for comparing relative changes in Cu coordination environments across matrices, while the fitted fractions are still interpreted semiquantitatively rather than as absolute phase fractions.

**Figure 4:**
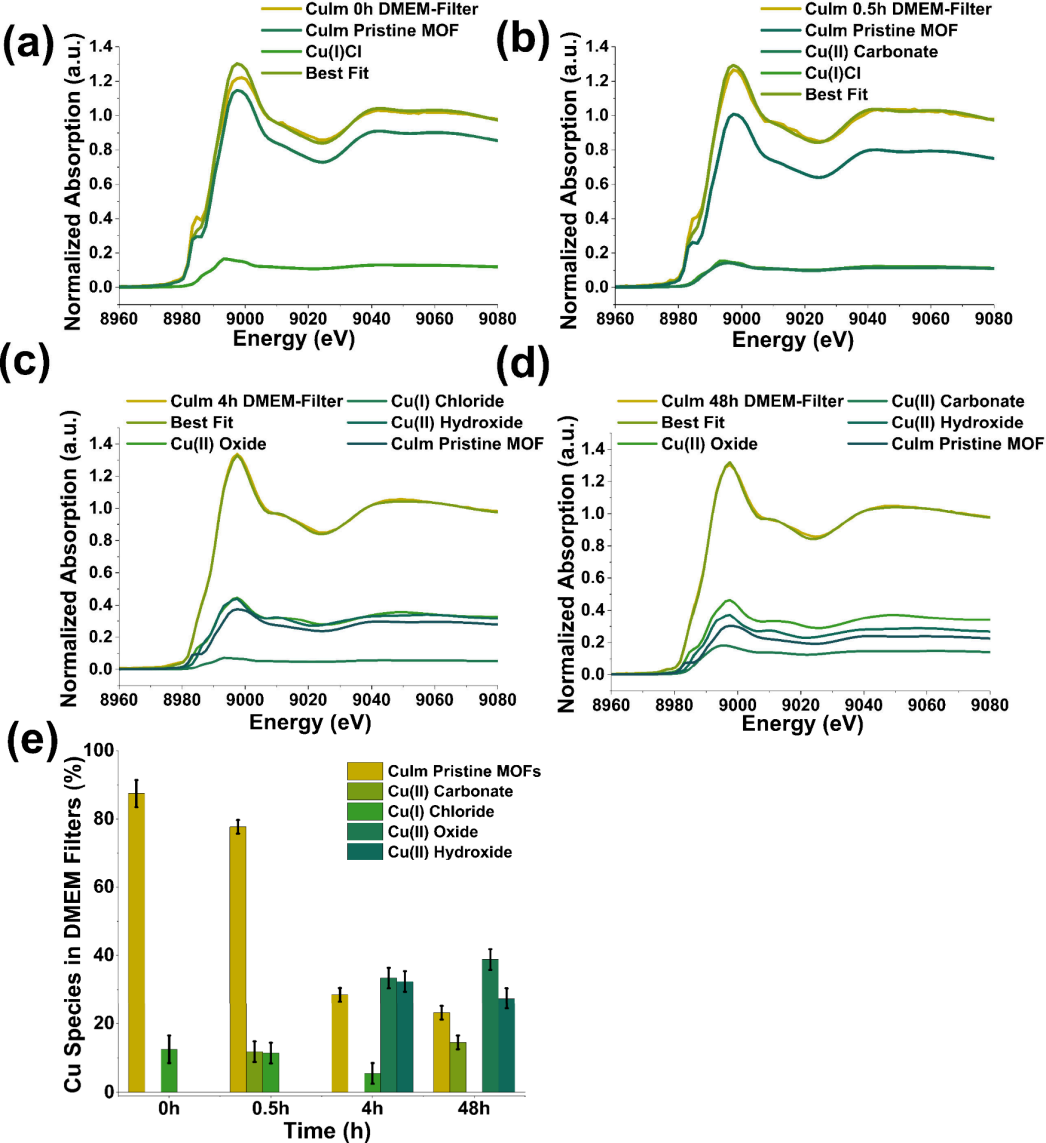
Linear combination fitting of particle-associated Cu speciation on filters during aging in serum-free DMEM. Cu K-edge XANES LCF for filter-retained solids collected during CuIm aging in serum-free DMEM. (a–d) Measured spectra (black) and LCF best fit (red) for representative time points (0 h, 0.5 h, 4 h, 48 h) with contributing reference spectra (e.g., pristine CuIm, Cu­(I) chloride, Cu­(II) carbonate, Cu­(II) oxide, Cu­(II) hydroxide as indicated). (e) LCF-derived fractions (%) versus time, showing rapid loss of CuIm-like coordination and emergence of multiple non-CuIm-like Cu coordination motifs by early time points, consistent with ligand-driven restructuring in a physiological, protein-free matrix. These motifs are assigned as spectral analogues of local Cu environments and are not taken alone as proof of bulk crystalline phase formation. Corresponding LCF fit-quality parameters, including χ^2^ and reduced χ^2^ values for each fitted spectrum, are provided in Table S3.

The LCF analysis shows that aging in ASW promotes a progressive shift in the average Cu local coordination environment toward hydroxide-like and chloride-associated spectral motifs (Figure 2). At 0 h, the filter-retained fraction is fitted as 79.5% CuIm-like, with minor oxide-like (10.3%) and chloride-associated (10.1%) Cu contributions (Figure 2a). By 4 h, the CuIm-like contribution decreases to 64.8%, while oxide-like and hydroxide-like motifs account for 13.9% and 12.1%, respectively (chloride-associated: 9.0%) (Figure 2b). By 48 h, the CuIm-like contribution decreases further to 53.1%, while the hydroxide-like contribution rises to 36.3% (chloride-associated, 8.6%; oxide-like, 1.8%) (Figure 2c,d). These values should not be read as evidence of stoichiometric crystalline CuO or Cu­(OH)_2_ formation. Rather, they provide a semiquantitative description of the gradual redistribution of Cu coordination environments within the retained particle-associated material. This is the quantitative counterpart of the EXAFS second-shell evolution in ASW (Figure 1d), consistent with slow salt-driven conditioning, surface/edge restructuring, and medium-range reorganization rather than a single bulk phase conversion. BHW (Figure 3): conservative trajectory with minor heterogeneity is shown. CuIm-like coordination remains the dominant contribution across all assessed time points (72.8% at 1 h; 67.8% at 24 h; 67.8% at 72 h), with only minor non-CuIm-like contributions distributed between chloride-associated and hydroxide-like motifs (Figure 3a–d). These LCF changes should not be overinterpreted as extensive phase transformation, but they are consistent with limited surface/defect-level heterogeneity under BHW exposure. Together with the XPS increase in surface Cu and O, the LCF data indicate that BHW is conservative at the dominant Cu-center level but not completely chemically inert at the surface. Serum-free DMEM produces the most rapid redistribution of Cu coordination motifs (Figure 4). At 0 h, the particle-associated fraction is dominated by a CuIm-like spectral contribution (87.4%), with a minor chloride-associated contribution (12.5%) (Figure 4a). By 0.5 h, the CuIm-like contribution decreases to 76.8% and a carbonate-associated motif appears (11.4%), alongside a chloride-associated contribution (11.7%) (Figure 4b). By 4 h, coinciding with convergence of the filter XANES spectrum toward the final-powder spectrum in Figure 1e, the CuIm-like contribution decreases to 28.5%, while oxide-like (33.4%) and hydroxide-like (32.4%) Cu motifs become dominant (Figure 4c). At 48 h, the particle-associated fraction remains enriched in non-CuIm-like motifs, including oxide-like, hydroxide-like, and carbonate-associated spectral contributions (Figure 4d,e). We interpret this as rapid node-level chemical redefinition and coordination-environment redistribution, not as unambiguous formation of separate crystalline CuO/Cu­(OH)_2_ phases. The persistence of CuIm-like PXRD and FTIR signatures at the end point indicates that local Cu-center restructuring can occur while sufficient long-range framework order and linker fingerprints remain detectable.

### Cu Mobilization into the <0.02 μm Dissolved/Complexed/Ultrasmall-Colloidal Fraction

ICP-MS analysis of Cu in the <0.02 μm filtrate adds a quantitative mass-transfer constraint for interpreting Cu mobilization from the retained particle-associated fraction. Because 0.02 μm filtration provides an operational rather than absolute molecular separation, this filtrate is interpreted here as the <0.02 μm dissolved/complexed/ultrasmall-colloidal Cu fraction, rather than as purely ionic dissolved Cu. It may therefore include Cu ions, Cu–ligand complexes, hydrolyzed/polynuclear Cu species and any Cu-bearing species smaller than the nominal filter cutoff. This definition is important because the objective of the present study is to compare the retained particle-associated Cu coordination environment with the Cu pool mobilized into the <0.02 μm fraction, not to claim complete separation between ionic and ultrasmall colloidal Cu. The dissolution/complexation curves (Figure S3) follow an asymptotic approach-to-plateau profile fitted with *y*(*t*) = *y*_0_ + *A* exp­(*R*_0_ *t*) (equivalent to an exponential approach-to-plateau form), indicating broadly comparable approach behavior but strongly different plateau concentrations.^18^

The dissolution/complexation profiles were fitted using an asymptotic exponential model:

$$y\left(t\right)=y_{0}+A\exp\left(R_{0}t\right)$$

where *y*(*t*) is the concentration of Cu measured in the <0.02 μm filtrate (μg mL^–1^) at time *t* (h), *y*_0_ is the fitted asymptote (plateau concentration), *A* is the pre-exponential amplitude, and *R*_0_ (h^–1^) is the apparent approach rate constant (negative for an approach-to-plateau). With *A* < 0and *R*_0_ < 0, this formulation is mathematically equivalent to an exponential approach-to-plateau form, *y*(*t*) = *y*_∞_[1 – exp­(−*kt*)], where *y*_∞_ = *y*_0_ and *k* ≈ −*R*_0_.

The plateau dissolved/complexed Cu pool differs by nearly an order of magnitude: 39.54 ± 4.14 μg mL^–1^ in serum-free DMEM (72 h), compared with 6.06 ± 1.01 μg mL^–1^ in BHW (72 h) and 4.04 ± 0.77 μg mL^–1^ in ASW (48–72 h) (Figure S3). This difference aligns directly with the LCF trajectories: DMEM is the matrix in which the solid rapidly becomes dominated by non-CuIm motifs (Figure 4c–e) and simultaneously sustains the largest dissolved/complexed Cu pool, consistent with ligand stabilization pulling Cu away from Cu–imidazolate coordination. In ASW, by contrast, the dissolved Cu pool remains small even as hydroxide-like contributions grow in the particle-associated fraction (Figure 2c,d), consistent with salt-driven surface conditioning and limited net mobilization.

A particularly strong mechanistic point emerges comparison of the time scales shows that: the particle-associated Cu-center environment in DMEM reaches its end-state signature by ∼4 h (Figure 1e; Figure 4c), while the dissolved pool continues rising toward its 72-h plateau (Figure S3). Thus, node reprogramming precedes completion of mobilization-meaning “rapid transformation” is not synonymous with rapid full dissolution. This decoupling is a hallmark of transformation trajectories and reinforces why early-time, node-resolved measurements are required.

XANES-LCF applied to serum-free DMEM filtrates shows that the dissolved/complexed Cu pool is itself chemically dynamic (Figure S5), evolving over the same early window in which the particle-associated fraction is being reprogrammed (Figure 4). The emergence of phosphate- and carbonate-associated motifs by 4 h in the filtrate (Figure S5) is chemically coherent with the rapid appearance of carbonate and hydroxide/oxide-like motifs in the filter-retained solids (Figure 4b,c) and with the steep rise in dissolved Cu concentration during the same period (Figure S3). Together, Figures 4, S3, and S5 demonstrate coupled solid–liquid evolution: the system moves through a sequence of time-dependent speciation mixtures rather than transitioning directly from CuIm to a single end product.

Liquid-phase XANES-LCF could be performed only for the serum-free DMEM filtrates because this matrix generated a sufficiently high <0.02 μm Cu concentration to obtain good signal-to-noise ratio at the Cu K-edge XANES. In contrast, the BHW and ASW filtrates produced Cu concentrations that were measurable by ICP-MS but too low for acquiring Cu K-edge XANES spectra in the liquids under the available measurement conditions. Therefore, the absence of corresponding BHW and ASW filtrate LCF should not be interpreted as the absence of <0.02 μm Cu species; rather, it reflects the lower mobilized Cu pool in these matrices. In the DMEM filtrate, the presence of oxide-like, hydroxide-like, chloride-like, phosphate-associated and carbonate-associated LCF motifs indicates that the <0.02 μm fraction contains chemically diverse Cu-bearing species. These motifs may arise from Cu–ligand complexes, hydrolyzed/polynuclear Cu species and/or ultrasmall Cu-bearing colloids smaller than the nominal 0.02 μm filter cutoff. Accordingly, Figure S5 reinforces the operational definition of the filtrate as a mobilized <0.02 μm Cu fraction, not as a purely ionic dissolved Cu pool.

### Linking End Point Characteristics with Time-Resolved Transformation

The end point (72 h) powders provide an important bridge between the time-resolved Cu K-edge XAS results (which constrain local Cu-center identity) and the complementary bulk/surface characterization (which probes long-range order and outer-surface chemistry). As shown in the time-resolved XAS series and LCF analysis (Figures 1–4), CuIm follows a matrix-dependent hierarchy in which BHW is largely conservative, ASW evolves gradually, and serum-free DMEM exhibits rapid early-time reprogramming that converges to the end-state by ∼4 h. We therefore use the end-point data set below (Figures 5 and 6; Figures S2–S6) to test whether framework-like structural signatures can persist while Cu-centered and surface chemistry diverge across matrices.

**Figure 5:**
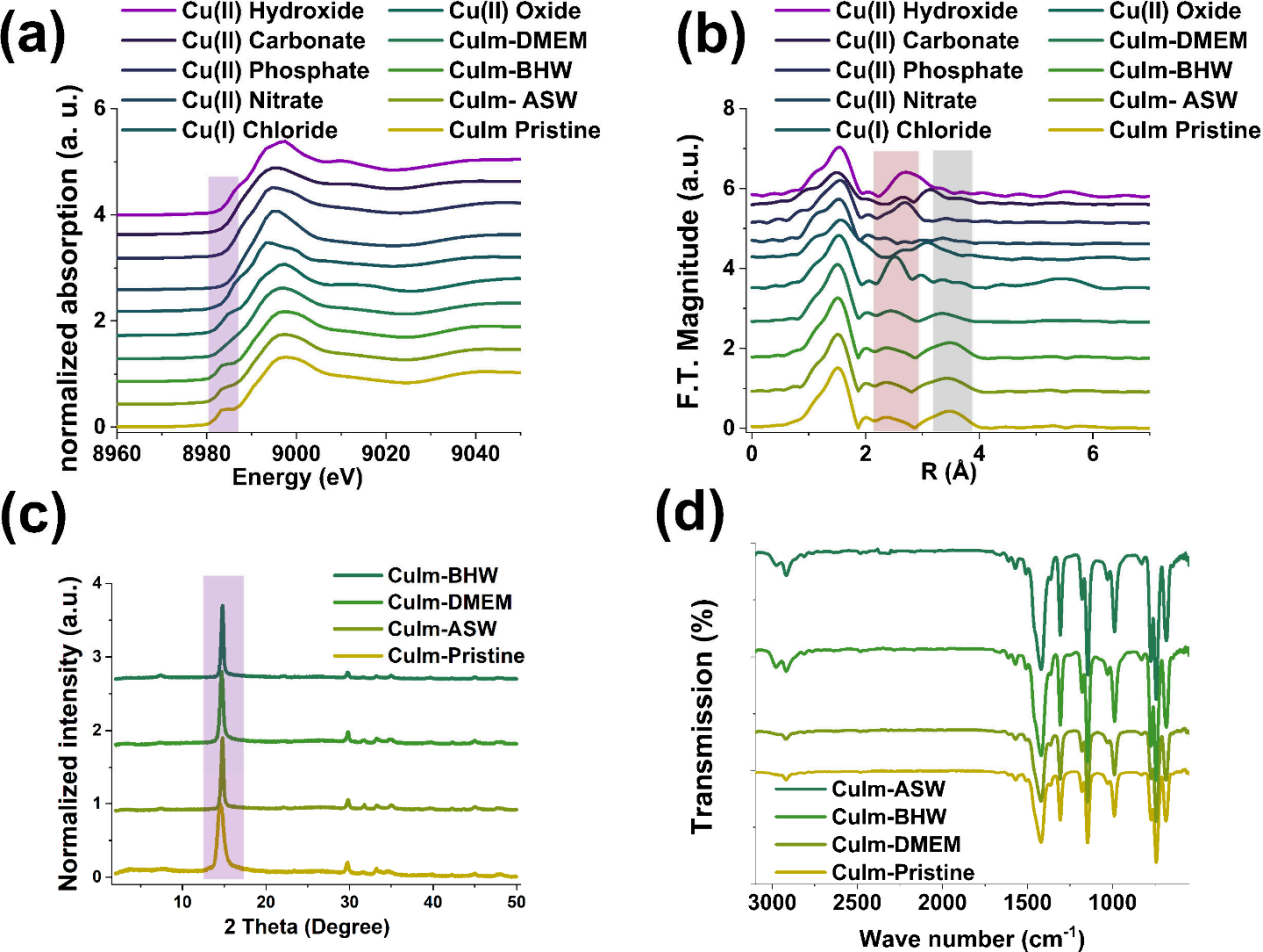
End-point (72 h) multiscale characterization of CuIm solids after aging in BHW, ASW, and serum-free DMEM. (a) Normalized Cu K-edge XANES and (b) Fourier transform magnitude of *k*-weighted EXAFS for pristine CuIm and 72 h end-point (“final powder”) solids recovered after aging in borehole water (BHW), artificial seawater (ASW) and serum-free DMEM, shown alongside representative Cu reference compounds used for speciation interpretation (Cu­(I) chloride; Cu­(II) nitrate, carbonate, phosphate, hydroxide, and oxide). Spectra are vertically offset for clarity and highlight matrix-dependent differences in the local Cu electronic structure and coordination environment at the end point. The highlighted regions indicate the principal spectral features used to compare matrix-dependent changes relative to pristine CuIm. (c) Powder X-ray diffraction (PXRD) patterns for pristine and aged solids (offset for clarity), showing retention of the dominant CuIm reflections after 72 h across all matrices, consistent with preservation of long-range order despite node-level chemical evolution. (d) FTIR spectra (600–3000 cm^–1^) of pristine and aged solids, showing persistence of the imidazolate vibrational fingerprint with matrix-dependent band reshaping, consistent with chemical conditioning of the framework environment following exposure.

**Figure 6:**
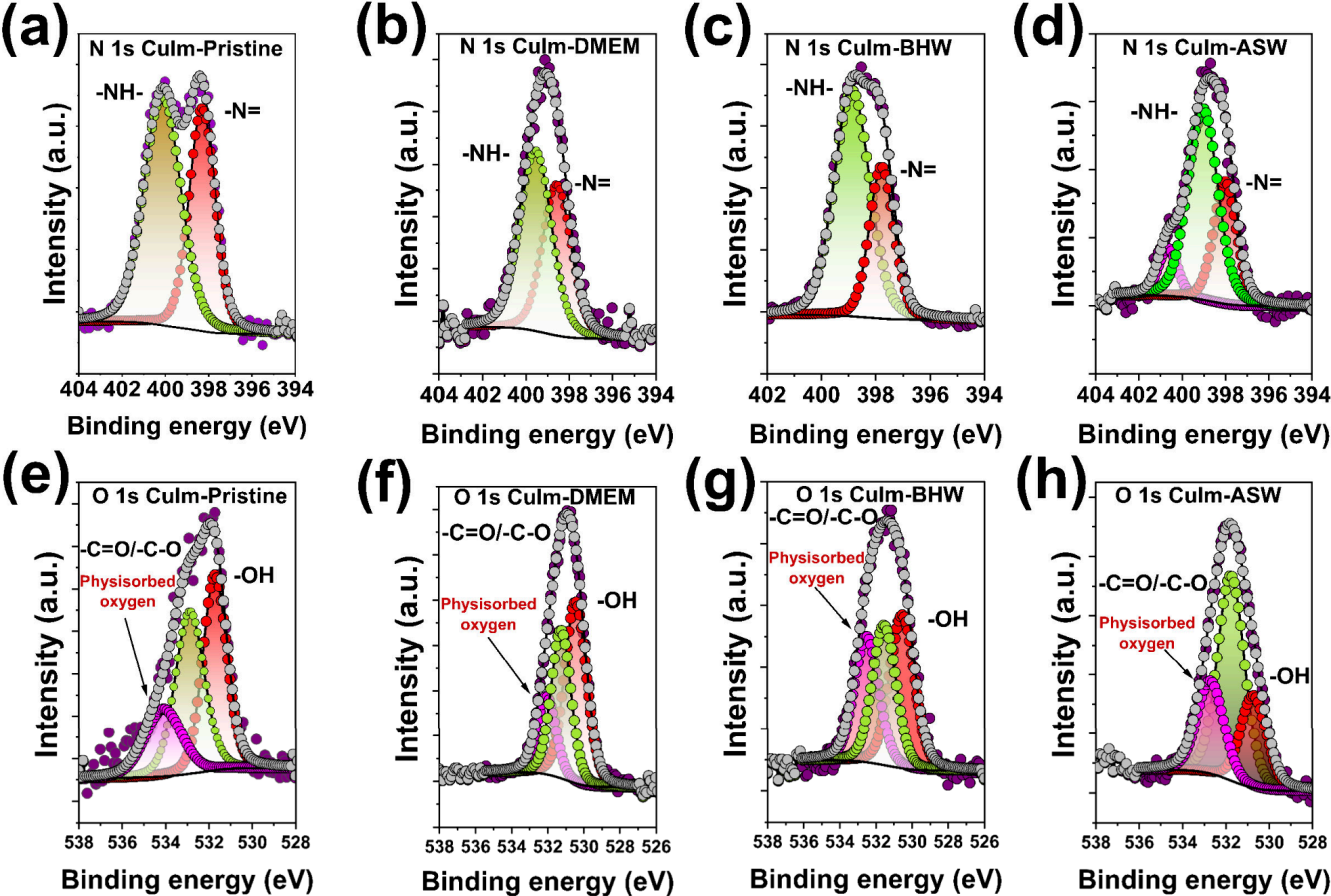
High-resolution XPS peak fitting of N 1s and O 1s regions for pristine and 72 h end point CuIm solids. High-resolution XPS core-level spectra and peak deconvolution for pristine CuIm and 72 h end point solids recovered after aging in borehole water (BHW), artificial seawater (ASW), and serum-free DMEM. (a–d) N 1s regions for CuIm-Pristine, CuIm-DMEM, CuIm-BHW, and CuIm-ASW, resolved into principal nitrogen environments consistent with imidazolate N motifs. (e–h) O 1s regions for CuIm-Pristine, CuIm-DMEM, CuIm-BHW, and CuIm-ASW, deconvoluted into oxygenated surface/near-surface environments, including hydroxylated/adsorbed oxygen, oxygenated carbon species, and physisorbed water where applicable. Together, these spectra show matrix-dependent surface chemical conditioning of CuIm after 72 h aging while complementing the bulk and local-structure assessments.

Against that kinetic backdrop, the end-point multiscale data set (Figure 5) directly demonstrates our core claim: structural persistence is decoupled from chemical persistence at the Cu center. Cu K-edge XANES/EXAFS of the final powders show matrix-dependent terminal coordination identities (Figure 5a,b) that are consistent with the time-resolved trajectories (BHW highly similar to pristine (Figure 3); ASW subtly modified; DMEM undergoes pronounced restructuring). Yet powder X-ray diffraction (PXRD) patterns retain the characteristic CuIm reflections across matrices (dominant low-angle peak near ∼14–15° 2θ and higher-angle features around ∼29–31° and ∼33–36° 2θ, extending toward ∼45–50° 2θ) (Figure 5c), indicating that the lattice does not undergo wholesale collapse into a fully amorphous product on the 72 h time scale.^13^ The practical implication is that the DMEM and ASW pathways can be dominated by local/medium-range restructuring, defect generation and heterogeneous coordination rewiring that strongly affects XAS (and reactivity/release), while leaving sufficient long-range order to appear stable by diffraction alone. This well-known pitfall–that XRD patterns may appear stable even as the underlying chemistry evolves–has been repeatedly highlighted in MOF stability reviews and underscores why stability must be defined operationally by both medium and metric. The practical implication is that structural persistence (PXRD) can coexist with chemical evolution at the metal center (XAS): the XAS changes seen in ASW and especially DMEM do not require a complete loss of crystallinity, because they can arise from local reorganization, defect formation, partial ligand exchange at accessible surface/edge sites, and/or increased disorder that primarily dampens higher-shell EXAFS contributions rather than destroying the entire lattice. This is fully consistent with the XAS observation that the first-shell contributions persist while second-/higher-shell features are the most sensitive to media-driven change.

The Fourier transform infrared (FTIR) spectra (Figure 5d) of the 72 h powders reinforce that conclusion, while also indicating medium-dependent surface/chemical conditioning. After 72 h aging, these imidazolate-derived bands remain clearly visible in all three end-point powders, again arguing against complete linker loss. However, the spectra also show systematic changes in relative intensity and band shape across matrices, which is consistent with a transformation in which the framework does not disappear but the outer coordination environment becomes progressively more heterogeneous and oxygen- or ion-associated. In ASW and BHW, the broad region around ∼3000–3600 cm^–1^ (data not shown) becomes more intense, which is assigned primarily to O–H stretching from adsorbed/interfacial water, hydrogen-bonded hydroxyl groups and hydrated/oxygenated surface species retained after exposure and drying. A contribution from N–H stretching or hydrogen-bonded imidazolate environments may also be present, but the stronger interpretation is surface hydration/oxygenation because XPS shows increased surface oxygen after aging, from 6.0 atom % in pristine CuIm to 9.0 atom % in BHW and 18.8 atom % in ASW (Table S1). In addition, the mid-IR region (roughly ∼1500–1000 cm^–1^) becomes comparatively more structured/intense, consistent with stronger contributions from oxygenated/ionic surface species (for example, carbonate-/phosphate-/sulfate-like vibrations in this window are common in salt-conditioned solids), whereas the DMEM-aged powder shows comparatively muted/redistributed intensity across the same region- consistent with a ligand-rich medium producing a reorganized, more disordered surface coordination motif rather than simply depositing a single dominant salt phase. Importantly, this FTIR pattern matches the XAS kinetic hierarchy: DMEM drives the fastest and most pronounced change at the Cu centers, and FTIR is consistent with that change being chemically subtle (coordination/surface chemistry) rather than a clean conversion to a completely different crystalline phase. Raman spectra of pristine and 72 h aged CuIm solids were also collected to complement the FTIR/PXRD analysis (Figure S8). The principal imidazolate-associated Raman bands are retained after aging in BHW, ASW and serum-free DMEM, supporting persistence of the framework/linker fingerprint.^19,20^ Matrix-dependent intensity and band-shape changes are consistent with surface conditioning and local coordination reorganization, reinforcing that framework-like vibrational signatures can persist even when Cu-centered XAS reveals chemical transformation.

The XPS survey spectra (Figure S2) and quantified surface atomic concentrations provide the most direct link to the coordination changes inferred by XAS. Pristine CuIm is linker-rich at the surface (C 70.2 atom %, N 21.8 atom %) with minor O (6.0 atom %) and a low apparent surface Cu signal (1.8 atom %), consistent with an imidazolate-terminated outer surface that partially shields Cu nodes within the top few nanometres (Table S1). After 72 h exposure, all matrices increase the apparent surface accessibility of Cu and enrich oxygen to varying degrees. The largest surface Cu enrichment is observed in ASW (Cu 4.2 atom %), followed by BHW (Cu 3.6 atom %) and DMEM (Cu 2.4 atom %), while oxygen rises most strongly for DMEM (O 19.0 atom %) and ASW (O 18.8 atom %), compared with BHW (O 9.0 atom %). Although BHW remains the most conservative matrix in the Cu K-edge XAS time series, the increase in surface Cu and O demonstrates that it still induces measurable surface conditioning, likely through hydration/oxygenation of accessible Cu sites and slight enrichment of Cu at the near-surface region. This reconciles the XAS and XPS data sets: the dominant bulk/average Cu coordination environment remains CuIm-like, while the outer surface undergoes modest chemical reorganization. This decoupling is informative: DMEM produces the strongest and fastest local restructuring in XAS, yet ASW yields the highest surface Cu atomic percentage by XPS. A coherent interpretation is that DMEM rapidly perturbs the local coordination environment at Cu (XAS) through ligand-driven exchange/rehybridization at coordination-active sites, whereas ASW (high ionic strength) promotes stronger surface exposure/heterogeneity and adsorption-driven reorganization that manifests as elevated surface Cu signal and broadened/perturbed N environments, without necessarily producing the same “early commit point” kinetics seen in DMEM.

High-resolution XPS peak positions support this matrix-specific coordination picture while arguing against clean formation of a bulk-like surface Cu–O phase detectable by XPS, even though XANES-LCF resolves oxide-like spectral contributions within the evolving Cu coordination environment (Figure 6). The N 1s envelope of pristine CuIm contains two components at 398.3 and 400.1 eV, assigned to imidazolate N in distinct environments (Cu-coordinated/sp^2^-like −N= and more protonated/hydrogen-bonded −NH– character) (Figure 6a–d). Upon exposure, DMEM shifts these components modestly to 398.5 and 399.5 eV, consistent with surface-level electron-density redistribution/coordination perturbation rather than complete loss of imidazolate chemistry. ASW shows the strongest N perturbation, with components at 398.01 and 399.04 eV and an additional higher-binding-energy contribution at 400.06 eV, consistent with a more positively polarized/hydrogen-bonded nitrogen environment stabilized under high ionic strength. In contrast, BHW remains closest to pristine, with the two components shifted to 397.7 and 398.8 eV, consistent with enhanced solvation but minimal chemical redefinition of the imidazolate environment. The O 1s region is especially important for connecting XPS to XAS and PXRD (Figure 6e–h). Across all samples, O 1s components occur exclusively above 530 eV. In pristine CuIm, the dominant O 1s component is at 531.7 eV, with higher-binding-energy contributions at 532.8 and 534.1 eV (commonly associated with hydroxylated surfaces, adsorbed water, and weakly bound oxygen-containing species).^21,22^ Critically, the absence of any contribution below 530 eV argues against formation of a bulk-like lattice Cu–O phase (i.e., it is not behaving like a clean conversion to CuO/Cu­(OH)_2_ at the surface within XPS detection). After aging, the main O 1s peak shifts to 530.7 eV in DMEM and 531.15 eV in BHW (stronger hydrogen bonding/enhanced hydration), whereas ASW retains a higher main component at 531.8 eV with additional oxygen components (e.g., ∼531.76 eV and 532.7 eV) consistent with multiple coexisting oxygenated/adsorbed environments under saline conditions. This is exactly the type of oxygen-rich but not oxide-lattice signature that reconciles all data sets: PXRD can remain CuIm-like, XAS can show coordination-shell disorder/reorganization, and XPS can show strong oxygen enrichment without requiring a dominant crystalline copper oxide product.

Finally, the Cu 2p spectra (Figure S2b) remain in the Cu­(II) regime across all conditions: pristine CuIm exhibits a Cu 2p_3_/_2_ feature at ∼934.8–935.2 eV with characteristic shakeup satellites in the ∼940–945 eV range, and the exposed samples retain Cu 2p_3/2_ within the Cu­(II) binding-energy window, supporting the conclusion that the dominant process is coordination/environmental reorganization rather than Cu redox or massive oxide precipitation.^23^ The reported broadening/intensity changes (strongest in ASW and DMEM) align tightly with the XAS results: environments that perturb higher-shell EXAFS and near-edge shoulders also produce greater surface heterogeneity and redistribution of N/O coordination signatures in XPS.

Taken together, the end point PXRD/FTIR/XPS provide a coherent mechanistic closure to the time-resolved XAS story. BHW supports a conservative transformation pathway rather than absolute chemical invariance: the dominant Cu-center identity remains XAS-similar to pristine CuIm, and the end powder remains linker-consistent, but XPS and LCF reveal modest oxygen enrichment, increased apparent surface Cu accessibility and minor non-CuIm-like coordination heterogeneity. ASW drives slow conditioning: the framework remains largely crystalline by PXRD and linker-bearing by FTIR, but both XAS and XPS show measurable, progressive second shell/surface heterogeneity consistent with ion-driven reorganization. Serum-free DMEM produces the key “rapid transformation” claim: the Cu-center environment begins changing within 1 min and reaches a stable end state by ∼4 h (filter spectra matching the 72 h powder), while the end powder still retains recognizable CuIm features by FTIR/PXRD but shows strong oxygen enrichment and N redistribution by XPS—precisely the signature of rapid, ligand-driven coordination restructuring that can be missed by end-point crystallinity alone.

XANES-LCF of the final powders (Figure S4) provides a succinct semiquantitative closure consistent with the trajectory story. BHW remains overwhelmingly CuIm-like (93.5%), ASW remains largely CuIm-like (81.3%) with measurable non-CuIm-like local motifs, while DMEM is dominated by oxide-like and carbonate-associated spectral contributions, with the CuIm-like contribution reduced to 18.8%. These values describe the terminal distribution of Cu local coordination environments within the recovered solids and should not be interpreted as direct crystallographic phase fractions. This interpretation is consistent with PXRD/FTIR retention of CuIm-like framework signatures and XPS evidence for oxygen-rich surface conditioning rather than wholesale conversion to a single-crystalline copper oxide or hydroxide product. High-angle annular dark-field scanning transmission electron microscopy (HAADF-STEM)-energy dispersive X-ray spectroscopy (EDS) (Figure S6) provides nanoscale support for matrix-dependent association of medium-derived elements (e.g., Cl-associated signatures in ASW and P/S association consistent with DMEM components), consistent with the emergence of chloride- and phosphate/carbonate-associated coordination motifs detected by LCF for solids and liquids (Figures 2–4, Figure S5) and with the strong O enrichment in XPS (Figure 6).

## Conclusion and Outlook

This study shows that nanoscale CuIm does not exhibit a single, medium-independent stability state; instead, it follows matrix-selected, time-dependent transformation trajectories that are only revealed when the Cu-center chemical identity is tracked directly. *Ex situ* time-resolved Cu K-edge XAS of the particle-associated fraction establishes a clear kinetic hierarchy (Figure 1): CuIm follows a conservative BHW trajectory in which the dominant Cu-centered XAS signature remains close to pristine over 72 h, undergoes slower restructuring in ASW that is expressed most strongly in medium-range EXAFS features, and transforms rapidly in serum-free DMEM, where Cu-center changes are detectable within 1 min and the particle-associated spectrum converges to the 72 h end-state by ∼4 h (Figure 1e,f).

LCF demonstrates that these behaviors are not binary (“changed/unchanged”) outcomes but continuous redistributions of Cu environments with measurable shifts at each sampled time point, particularly in ASW and DMEM (Figures 2–4). In ASW, the particle-associated fraction evolves from 79.5% CuIm at 0 h to 53.1% CuIm at 48 h, accompanied by a pronounced growth of hydroxide-like motifs (36.3% at 48 h) (Figure 2d). In serum-free DMEM, CuIm decreases from 87.4% at 0 h to 28.5% at 4 h, with oxide- and hydroxide-like motifs dominating by the same time point (Figure 4c,e), consistent with rapid redistribution of Cu coordination environments in a ligand-rich, protein-free DMEM matrix.

Solution-phase measurements provide the necessary mass-transfer constraint to interpret these speciation changes as exposure-relevant transformation rather than purely spectral drift. Cu mobilization into the <0.02 μm fraction is strongly medium dependent (Figure S3), approaching plateaus of 39.54 ± 4.14 μg mL^–1^ (DMEM), 6.06 ± 1.01 μg mL^–1^ (BHW), and 4.04 ± 0.77 μg mL^–1^ (ASW), while fitted approach rates are broadly similar. Importantly, the coupled solid–liquid data set reveals two distinct time scales: in DMEM, Cu-center reprogramming in the solid fraction reaches its end-state by ∼4 h (Figures 1e and 4c), whereas the dissolved/complexed Cu pool continues to rise toward its plateau thereafter (Figure S3). Liquid-phase XANES-LCF further shows that the dissolved Cu pool is itself chemically dynamic over the same early window (Figure S5), reinforcing that the system progresses through time-dependent speciation mixtures rather than switching directly from CuIm to a single terminal product.

Crucially, the end-point characterization demonstrates the manuscript’s central decoupling: framework-like structural signatures can persist while node and surface chemistry are substantially rewritten. After 72 h, PXRD and FTIR retain CuIm-like reflections and imidazolate fingerprints across matrices (Figure 5), yet XPS quantifies strong, matrix-dependent surface conditioning that tracks the Cu-center hierarchy (Figure 6; Table S1; Figure S2). Pristine CuIm is C/N rich at the surface (C 70.2 atom %, N 21.8 atom %) with low O (6.0 atom %), whereas aged powders show pronounced oxygen enrichment, reaching O 19.0 atom % in DMEM and O 18.8 atom % in ASW (Table S1). In parallel, final-powder LCF provides quantitative closure consistent with the time-resolved trajectories: BHW remains overwhelmingly CuIm-like (93.5%), ASW remains largely CuIm-like (81.3%) with secondary motifs, while DMEM ends dominated by secondary motifs (Cu­(II) oxide 47.2%, Cu­(II) carbonate 33.9%) with CuIm reduced to 18.8% (Figure S4). Together, these results show why end-point crystallinity alone can generate false stability calls: the exposure-relevant identity of CuIm is defined by early-time trajectory chemistry, not only by whether diffraction peaks remain visible. Because these conclusions are derived from ex situ, operationally separated fractions, transient weakly bound species and intermediate states may be under-represented in the recovered solids. Because the separation is based on a nominal 0.02 μm filter cutoff, Cu-bearing species smaller than this size may pass into the filtrate. Accordingly, the filtrate is interpreted as an operational <0.02 μm dissolved/complexed/ultrasmall-colloidal Cu pool. The DMEM filtrate XANES-LCF confirms that this pool contains chemically diverse Cu coordination environments, while BHW and ASW filtrates were too dilute for robust liquid XANES analysis. This limitation does not affect the central matrix comparison because the same fractionation protocol was applied consistently across all matrices and time points, and the retained particle-associated fraction and mobilized <0.02 μm Cu pool were analyzed separately. These results are summarized schematically in Figure 7, which illustrates how the same CuIm nanosheet MOF follows conservative, slow-reorganizing or rapid commit-point transformation trajectories depending on matrix chemistry.

**Figure 7:**
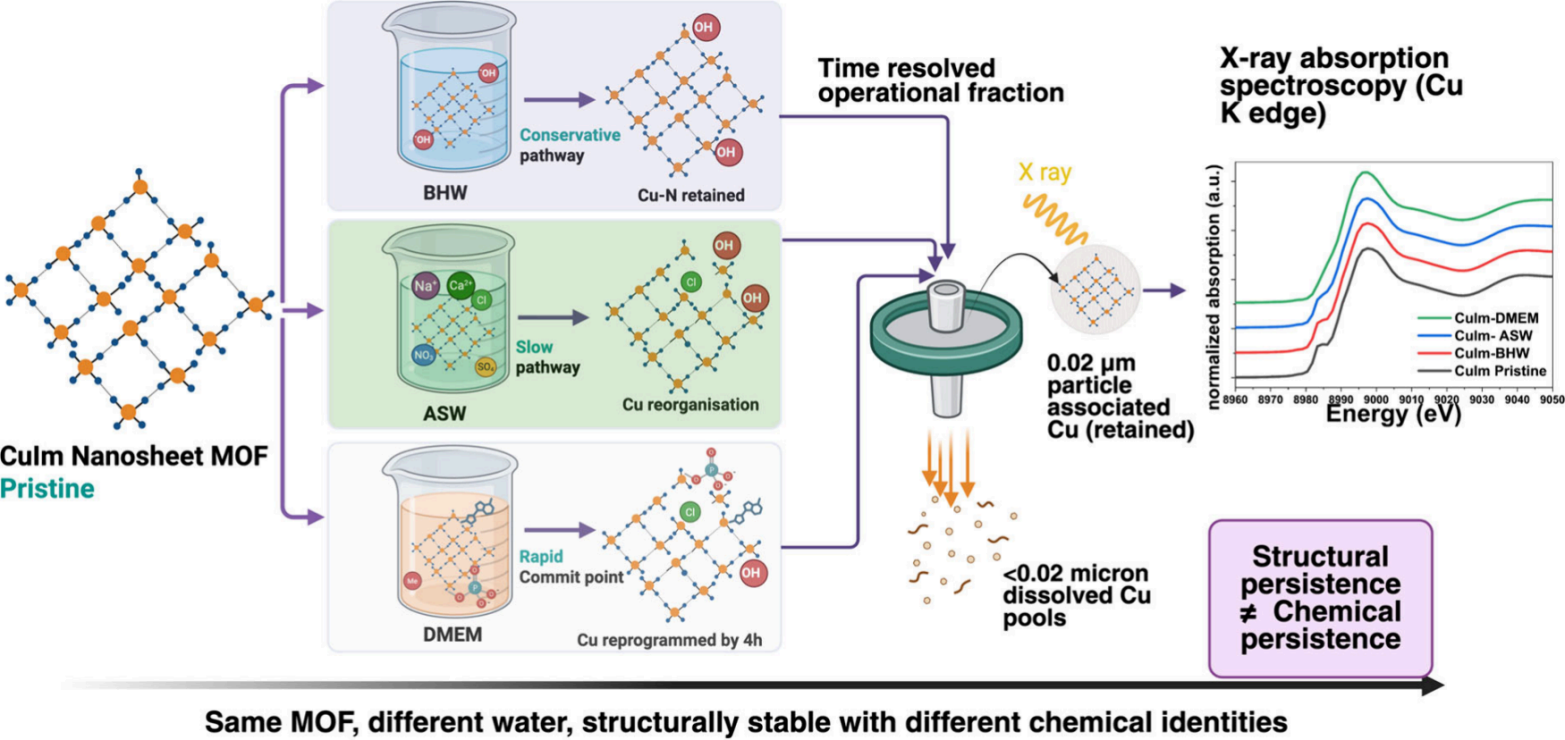
Graphical summary of matrix-selected transformation trajectories of CuIm nanosheet MOFs. Pristine copper–imidazolate (CuIm) nanosheets were exposed to borehole water (BHW), artificial seawater (ASW), and serum-free DMEM, producing distinct transformation pathways. BHW preserves the dominant Cu–N framework environment with only minor surface conditioning, ASW induces slower salt-driven Cu reorganization, and serum-free DMEM drives rapid Cu-center reprogramming by approximately 4 h. Time-resolved 0.02 μm operational fractionation separates the particle-associated Cu fraction from the mobilized <0.02 μm Cu pool, while Cu K-edge XAS/LCF resolves changes in local Cu identity. The schematic highlights the central conclusion that framework-like structural persistence can coexist with matrix-dependent chemical transformation at the Cu center. Created in BioRender. Chakraborty, S. (2026), https://BioRender.com/af6l6f9.

### Implications for MOF Stability Screening and SSbD

These findings reinforce that MOF stability must be treated as a function of medium composition, time scale, and metric, rather than as a single label.^24^ For coordination-active metals such as Cu, diffraction persistence can coexist with rapid node/surface rewriting in biologically relevant matrices, with direct consequences for performance claims and for safe-and-sustainable-by-design decisions: the relevant question is which coordination entity is delivered during the early exposure window that controls subsequent fate, release, and reactivity. A practical minimum data set for comparable reporting emerges from the coupled evidence here: early time node-resolved spectroscopy for the particle-associated fraction, an operational measure of mobilization into dissolved/complexed pools with clearly defined fractionation, and an orthogonal surface probe (e.g., XPS) to quantify conditioning.^25^

### Outlook: Why the Next Step Must Be *in Situ* Rapid Transformation Mapping

The most forward-looking insight from this data set is methodological: because speciation changes at essentially every sampled time point (especially in serum-free DMEM and ASW), key events almost certainly occur between filtration-resolved snapshots, and the order of early processes (ligand exchange, surface conditioning, dissolution/complexation, and secondary motif nucleation) cannot be reconstructed unambiguously ex situ. *Ex situ* workflows also impose operational fractionation (filtration, rinsing, drying) that can remove weakly bound species and relax transient states, biasing recovered solids toward apparently persistent motifs. The clear next frontier is therefore in situ rapid-mixing/medium-exchange experiments coupled to QEXAFS/Quick-XAS, enabling subsecond XANES and second-scale EXAFS acquisition during the decisive early window.^10,26,27^ Such approaches are explicitly designed to follow dynamic structural changes with millisecond-to-second time resolution in operando settings, providing the technical route to resolve the “commit point” chemistry implicated here.^25^

Finally, the framework established here makes the most informative follow-ups straightforward: targeted dissection of individual DMEM components (e.g., phosphate, bicarbonate, amino acids) to identify the dominant ligands driving rapid Cu reprogramming; extension to serum-containing media to test competition between protein adsorption and small-ligand complexation; concentration dependence of the commit point to connect mechanistic stress tests to realistic exposures; and coupling trajectory changes to functional/hazard end points to operationalize trajectory-informed screening.

## Experimental Section

### Materials

Copper nitrate and the imidazolate linker precursor were procured from Fisher Scientific (U.K.). All additional salts and precipitated standards used in this study (including reference copper compounds used for Cu K-edge XANES/LCF) were of analytical grade and purchased from Sigma-Aldrich, unless otherwise stated. Deionized water was used for solution preparation and rinsing steps. Three benchmark matrices were used to span an abiotic-to-biorelevant gradient (composition, pH and rationale summarized in Table S2): (i) borehole water (BHW) as a freshwater-like, carbonate/bicarbonate-containing hard-water matrix (typical pH ∼7.0–7.5), (ii) artificial seawater (ASW) as a marine-like high-ionic-strength matrix (pH ∼7.5), and (iii) DMEM high glucose as a physiological, ligand-rich matrix used without serum (typical pH ∼7.2–7.4) to isolate ligand-driven transformation pathways from protein-corona effects. For operational separation of particle-associated versus dissolved/complexed copper during time-resolved aging, 0.02 μm syringe filters (Cytiva, U.K.) were used throughout.

### Synthesis and Characterization of CuIm MOFs

Copper–imidazolate MOFs (CuIm) were synthesized by an aqueous precipitation route followed by low-temperature freeze-drying activation. Briefly, copper­(II) nitrate trihydrate (3.238 g) was dissolved in 24 mL of ultrapure water and 2-methylimidazole (6.638 g, mIm) was dissolved in 96 mL of ultrapure water (each stirred for 30 min). The copper precursor solution was then added dropwise to the ligand solution under continuous stirring and allowed to react at room temperature for 24 h, producing a brown precipitate. The solid was isolated by centrifugation and washed repeatedly with ultrapure water to remove residual reagents and soluble byproducts. The washed wet solid was frozen at −80 °C and subsequently lyophilized at −55 °C for 10–12 h to yield an activated, free-flowing CuIm powder, which was stored in sealed glass vials under dry conditions until use. The pristine CuIm was characterized by scanning electron microscopy (SEM) to assess morphology, powder X-ray diffraction (PXRD) to confirm phase identity/crystallinity, and FTIR spectroscopy to verify imidazolate linker signatures and framework bonding.

### Transformation Studies in Biological and Environmental Matrices

Time-dependent transformation studies of nanoscale CuIm MOFs were performed using an *ex situ* sampling design. Three benchmark matrices were selected to span an abiotic-to-biorelevant gradient and to decouple salt-driven from ligand-driven pathways: a freshwater-like groundwater matrix (borehole water, BHW), a marine-like high-salinity matrix (artificial seawater, ASW), and a physiological, ligand-rich but protein-free matrix (serum-free Dulbecco’s modified Eagle medium, DMEM). CuIm suspensions were prepared at 2 mg mL^–1^ in 50 mL of each matrix. This comparatively high particle loading was intentionally chosen to ensure sufficient particulate copper mass could be retained on 0.02 μm filters at each time point, thereby providing adequate signal-to-noise for downstream synchrotron-based measurements of the particle-associated fraction.

All exposures were conducted at room temperature under orbital shaking (120 rpm) for up to 72 h. To capture rapid early-time evolution in the biorelevant matrix, serum-free DMEM was sampled at 0 h, 1 min, 10 min, 30 min, 1 h, 4 h, 8 h, 24 h, 48 h, and 72 h. BHW was sampled at 0 h, 30 min, 1 h, 4 h, 24 h, 48 h and 72 h, and ASW at 0 h, 1 h, 4 h, 24 h, 48 h, and 72 h. Each matrix–time point condition was prepared in multiple independent replicates (typically *n* = 3, depending on the time point).

At each time point, 0.5 mL aliquots were withdrawn from the exposure vessel and immediately passed through 0.02 μm syringe filters (Cytiva, U.K.). This filter size was selected as a stringent operational cutoff to separate the parent nanoscale CuIm particles and larger transformed/aggregated Cu-bearing solids from the <0.02 μm Cu pool while preserving the rapid sampling needed for minute-to-hour transformation kinetics. The retained material is therefore defined as the particle-associated fraction, comprising CuIm and any transformed Cu-bearing solids retained by the membrane. The filtrate is operationally defined as the <0.02 μm dissolved/complexed/ultrasmall-colloidal Cu fraction, recognizing that it may include Cu ions, Cu-ligand complexes, hydrolyzed/polynuclear Cu species and any Cu-bearing colloids smaller than the nominal pore size. Filters containing retained particles were rinsed with deionized water to remove residual matrix components and minimize contributions from trapped salts/medium constituents, then dried at 60 °C overnight prior to analysis of the particle-associated fraction. The corresponding filtrates were collected for ICP-MS analysis (Nexion 350, PerkinElmer, USA). Liquid-phase Cu K-edge XANES was attempted for the filtrates; however, reliable XANES-LCF could only be obtained for serum-free DMEM filtrates because BHW and ASW filtrate Cu concentrations were too low for robust liquid-phase XAS under the available measurement conditions.

### *Ex Situ* Time-Resolved Transformation Studies Using Synchrotron X-ray Absorption Spectroscopy (XAS)

Cu K-edge X-ray absorption spectroscopy (XAS; *E*_0_ ≈ 8979 eV) was used to track time-dependent changes in the local coordination environment and electronic state of Cu in the particle-associated and dissolved fractions during aqueous aging of CuIm MOFs. XAS measurements were performed at the BAM*line* located at BESSY-II storage ring operated by Helmholtz Centre Berlin (HZB) in both transmission and fluorescence mode simultaneously.^28^ The incident energy was tuned by a double crystal monochromator in a Si(111) arrangement (delivering an intrinsic resolution of Δ*E*/*E* = 2 × 10^–4^). The measurements were done in continuous mode on-the-fly, where the DCM is moving without stopping, while the ZEBRA unit triggers data acquisition as the desired energy positions are reached. The energy range scanned comprised in total 400 eV, starting at −100 eV below the edge and ending at 300 eV above the edge (for XANES), total of 1000 eV, starting at −200 eV below the edge and ending at 800 eV above the edge (for EXAFS) and equidistant 0.2 eV energy steps. In case of transmission, the signal from the sample is extracted as logarithm of beam intensity ratio measured by first and a second ionization chambers (*I*_0_, *I*_1_), following the known Lambert–Beer law; a reference foil was placed between second and third ionization chamber (*I*_1,_ *I*_2_) and was measured simultaneously with the sample in transmission mode. For fluorescence, a 4-element silicon drift detector (SDD) was placed between *I*_0_ and the sample so that the fluorescence signal was collected in backscattered mode.

To follow time-dependent speciation in the aqueous phase, filtrates obtained at each time point were measured at the Cu K-edge using liquid sample holders with Kapton X-ray-transparent windows designed in Spectroscopy Group at Diamond Light Source. The cell geometry provided a 1 mm liquid thickness, with ∼80 μL of filtrate loaded per measurement. For these solution samples, Cu K-edge XANES was acquired to capture changes in Cu speciation and coordination symmetry in the dissolved/complexed fraction as aging progressed, providing a direct complement to the particle-associated measurements.

In addition to time-resolved aliquots, the residual solid material remaining in each exposure vessel after 72 h was recovered as an end point “final powder” for each matrix. These end-product solids were analyzed at the Cu K-edge (XANES and EXAFS) to characterize the terminal coordination/electronic state of Cu after prolonged exposure, enabling direct comparison between early-time transformation trajectories and the final transformed identity in each medium. To resolve how copper speciation evolved during aging, we applied linear combination fitting (LCF) to the Cu K-edge XANES using a curated library of reference spectra representing plausible Cu end members expected under the tested matrices. LCF was performed for all liquid filtrate time points to quantify time-dependent changes in the dissolved/complexed Cu pool, and for selected solid samples (filter-retained CuIm at key time points) together with the 72 h end-point (“final powder”) to assess whether the particle-associated fraction remained CuIm-like or transitioned toward secondary Cu motifs during exposure. The LCF analysis DMEM filtrates provided adequate Cu K-edge signal-to-noise; BHW and ASW filtrates were unsuitable for reliable liquid-phase LCF. The reference set comprised the pristine CuIm MOF alongside representative Cu­(I) and Cu­(II) compounds, including Cu­(II) carbonate, Cu­(II) citrate, Cu­(NO_3_)_2_ (Cu­(II) nitrate), Cu­(II) sulfide, Cu­(II) hydroxide, Cu­(I) chloride (CuCl), Cu­(I) sulfide, CuO, Cu­(II) phosphate, and Cu­(II) chloride (CuCl_2_). Measurements of the membrane filters were collected in transmission, and solution were measured in fluorescence mode (notably certain filter-mounted solids and liquid cells), to ensure adequate signal-to-noise without excessive absorber thickness effects.

A Cu foil standard was measured simultaneously for energy calibration, and all spectra were aligned to the foil reference prior to averaging. Each sample was scanned three times to improve the signal-to-noise ratio and to confirm spectral reproducibility.

XAS data evaluation and treatment were performed by using Larch: Data Analysis Tools for X-ray Spectroscopy.^29^ Repetitions of the same sample were merged and normalized for XANES evaluation and linear combinations fittings. LCF analysis were performed between 8950 and 9090 eV using the least-squares fitting method, forcing the weights of the components to sum to 1. EXAFS data correspond to Fourier transformed *k* ^2^χ­(*k*)-weighted convoluted with a Hanning-type window over the range 3 and 12.5 Å^–1^. Fit quality was evaluated using the χ^2^ and reduced χ^2^ values returned from the least-squares fitting output.

### Characterization of Final Powder of CuIm Post-Transformation Experiment

End-point (“72 h”) CuIm solids from BHW, ASW and serum-free DMEM were recovered from the remaining suspensions at the end of each transformation experiment, rinsed thoroughly with deionized water to remove residual salts/medium components, and dried prior to ex situ characterization. The dried powders were then analyzed using a complementary suite of bulk and nanoscale techniques (FTIR, PXRD, XPS, and HAADF–STEM/EDS) to resolve (i) retention or loss of long-range crystallinity, (ii) changes in surface chemical state and elemental composition, and (iii) evidence for matrix-derived elemental association or secondary-phase formation at the particle level. This “end-product” workflow mirrors the multitechnique approach used in our hierarchical transformation studies to connect atomistic coordination changes observed by XAS with bulk- and surface-sensitive signatures of transformation.

Fourier transform infrared (FTIR) spectra of pristine CuIm and 72 h end-point powders were recorded using a PerkinElmer Spectrum instrument over 600–4000 cm^–1^ (ATR mode). Samples were measured under identical acquisition settings to enable direct, semiquantitative comparison of changes in the imidazolate-associated vibrational bands and the emergence/broadening of features consistent with hydroxylation, carbonate/phosphate association, or adsorption of medium-derived components. PXRD patterns were collected on a Malvern Panalytical diffractometer to assess retention of CuIm crystallinity and to identify any new crystalline phases in the 72 h solids relative to the pristine material. Powders were gently packed onto low-background holders to minimize preferred orientation and scanned over an angular range sufficient to capture the principal CuIm reflections and potential secondary phases (typical scans up to ∼50° 2θ). Raman spectra of pristine CuIm and 72 h aged solids recovered from BHW, ASW and serum-free DMEM were collected in the 500–2000 cm^–1^ fingerprint region to complement FTIR analysis of the imidazolate framework/linker vibrations. Spectra were baseline-corrected and vertically offset/normalized for comparison of the principal vibrational features across pristine and aged solids.

XPS was used to quantify media-dependent changes in surface elemental composition and bonding environments after 72 h aging. Dried powders were mounted on conductive carbon tape and analyzed using survey scans to establish the surface elemental inventory, followed by high-resolution spectra of the core regions most diagnostic for CuIm transformation (Cu 2p, N 1s, and O 1s). Spectra were processed using consistent background subtraction and peak-fitting protocols across all samples to allow robust comparison of binding energy shifts, satellite structure (Cu 2p), and changes in N/O/C contributions indicative of ligand loss, hydroxylation, or secondary-phase formation.

HAADF–STEM coupled with energy-dispersive X-ray spectroscopy (EDS) was used to map nanoscale elemental distributions and identify colocalization patterns indicative of medium-dependent transformation products. STEM–EDS was performed on a Talos F200X G2 operated at 200 kV in STEM mode with HAADF imaging and EDS mapping. In addition to Cu, maps targeted elements expected either from the CuIm framework (C, N, O) or introduced/accumulated from the exposure matrices (e.g., Na, Cl, Mg, Ca, P, and S) to determine whether transformation produced spatially heterogeneous Cu-rich domains, surface-enriched layers, or co-precipitated/adsorbed matrix-derived species.

## Supplementary Material



## Notes

Additional experimental and characterization data, including baseline CuIm nanosheet characterization, XPS survey and Cu 2p spectra, Cu mobilization into the <0.02 μm fraction, end point and filtrate Cu K-edge XANES-LCF analyses, HAADF-STEM/EDS elemental mapping, schematic representation of the CuIm nanosheet framework, Raman spectra of pristine and aged solids, exposure matrix composition, XPS surface atomic composition, and LCF fit-quality parameters (PDF)
