# Supplementary material for: Structural Persistence Masks Commit-Point Chemical Transformation in Copper–Imidazolate Nanosheet Metal–Organic Frameworks
Source: ACS Nano. 2026 Jun 23;20(26):18984–98. doi: 10.1021/acsnano.6c06011 (PMC13348175; doi:10.1021/acsnano.6c06011)
Supplement: Supplementary file 1 [file nn6c06011_si_001.pdf]

Structural persistence masks commit-point chemical transformation in copper–imidazolate nanosheet metal-organic frameworks

Swaroop Chakraborty[a]*, Ana Guilherme Buzanich[b]*, Prathmesh Bhadane[c], Hiroto Kitaguchi[d], Sang Pham, Iuliia Mikulska[d]*

[a] School of Geography, Earth & Environmental Sciences, University of Birmingham, Edgbaston, B15 2TT, UK

[b]Bundesanstalt für Materialforschung und -prüfung (BAM), Richard-Willstätter-Straße 11, 12489 Berlin, Germany

[c] Materials Engineering, Indian Institute of Technology, Gandhinagar, 382355, India.

[d] Facility of Electron Microscopy, University of Birmingham, Edgbaston, B15 2TT, UK

[e] Diamond Light Source, Harwell Science and Innovation Campus, Didcot, OX11 0DE UK.

*Correspondence- s.chakraborty@bham.ac.uk; ana.buzanich@bam.de; iuliia.mikulska@diamond.ac.uk

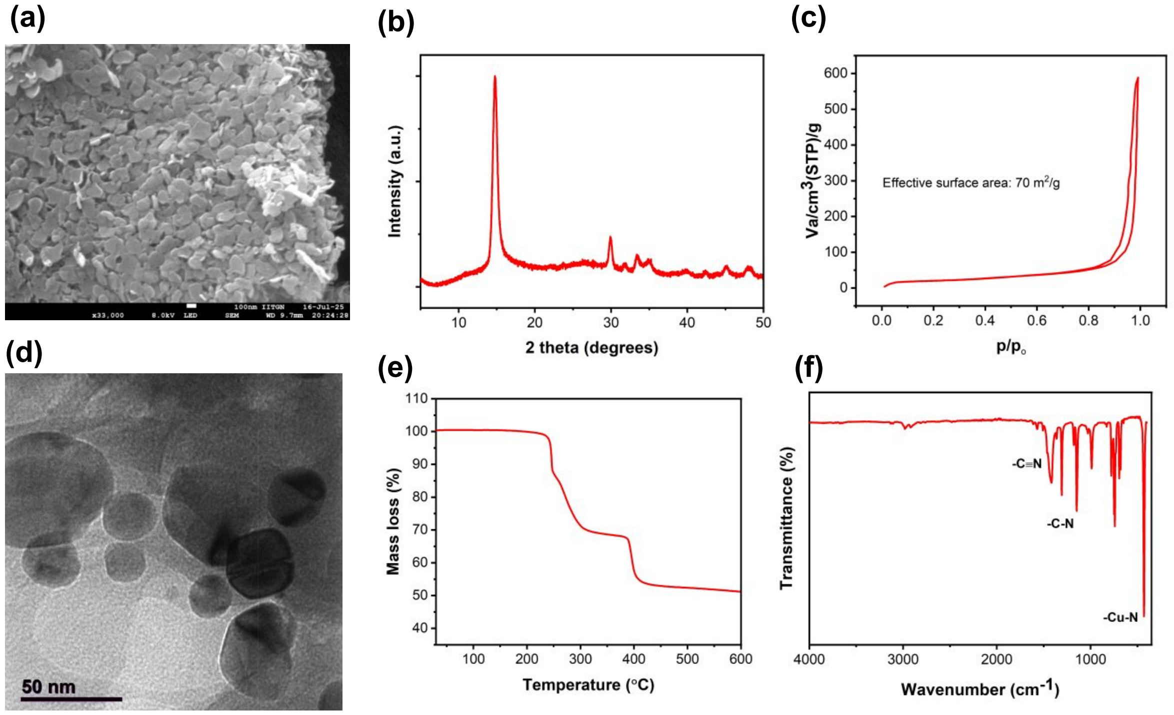

Figure S1. Baseline characterisation of the as-synthesised, freeze-dried CuIm MOFs. (a) SEM micrograph showing the agglomerated particle morphology of CuIm. (b) Powder X-ray diffraction (PXRD) pattern confirming the crystalline CuIm phase. (c) N_2 adsorption–desorption isotherm (77 K) used to estimate an effective surface area (~70 m^2 g^{-1}. (d) TEM image (50 nm scale bar) showing nanoscale CuIm particles and size/morphology heterogeneity. (e) Thermogravimetric analysis (TGA) trace showing multi-step mass loss with increasing temperature, consistent with removal of adsorbed species followed by framework decomposition. (f) FTIR spectrum (4000–600 cm^{-1}) showing the characteristic imidazolate linker fingerprint, including bands assigned to C=N/C–N vibrations and Cu–N coordination.

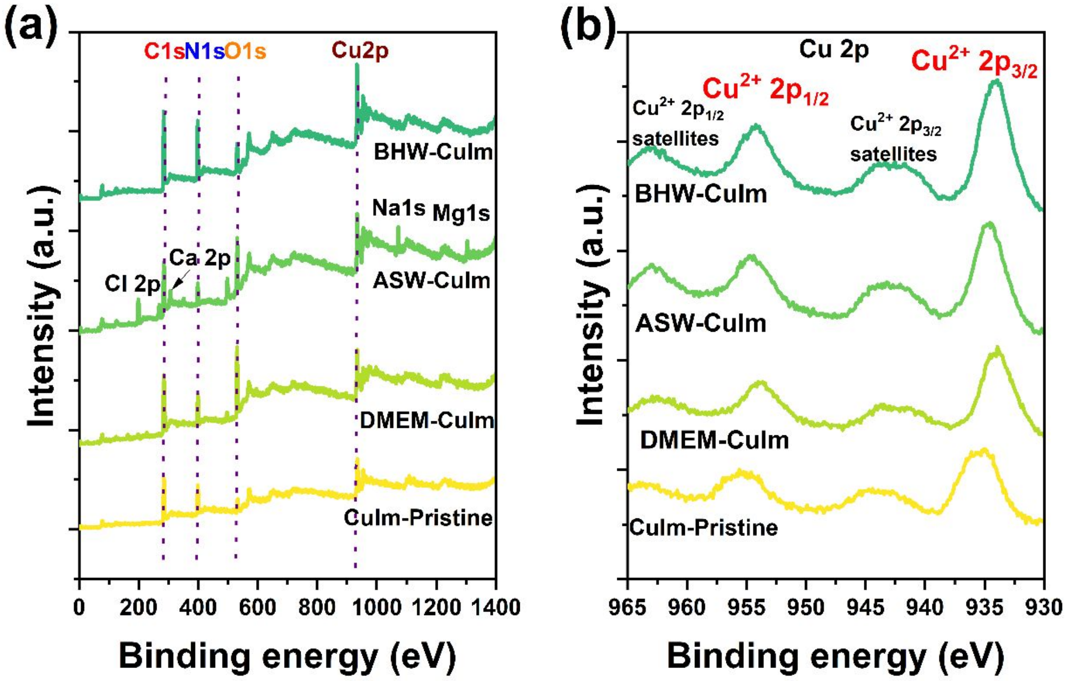

Figure S2. XPS survey and Cu 2p region for pristine and 72 h end-point CuIm powders **(a)** XPS survey spectra for pristine CuIm and 72 h end-point powders recovered after ageing in BHW, ASW, and serum-free DMEM, highlighting C 1s, N 1s, O 1s and Cu 2p regions; additional features associated with matrix-derived elements (e.g., Na, Mg, Ca, Cl) are indicated where present. **(b)** High-resolution Cu 2p region showing Cu(II) shake-up satellites and the Cu $2p_{3/2}/2p_{½}$ features for pristine and aged solids, supporting predominantly Cu(II)-type surface signatures across matrices.

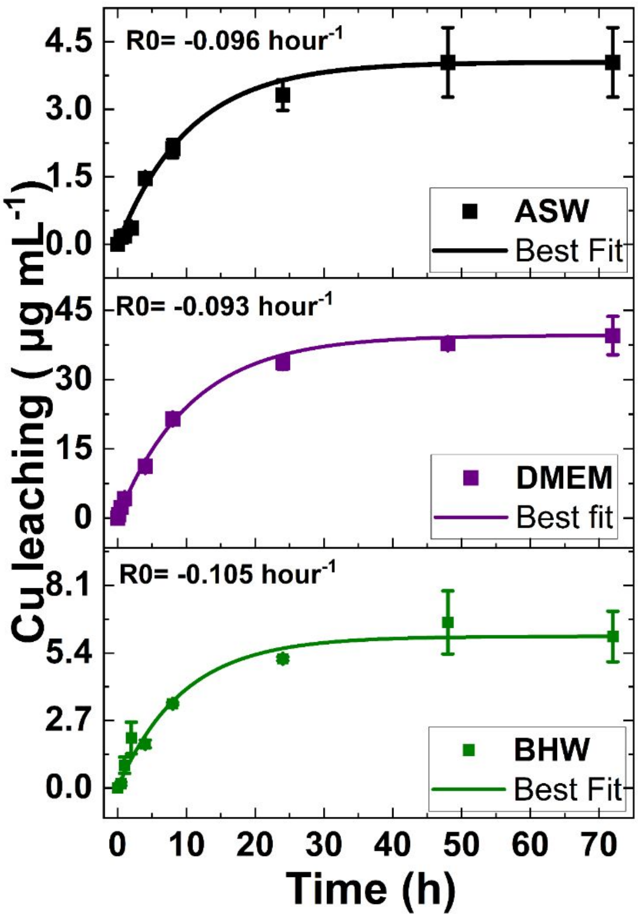

Figure S3. Cu mobilisation into the <0.02 μm dissolved/complexed/ultrasmall-colloidal filtrate fraction and exponential-to-plateau fitting. Time-dependent Cu concentration in the <0.02 μm filtrate quantified by ICP-MS for ASW, serum-free DMEM and BHW. The filtrate is operationally defined as the <0.02 μm Cu fraction and may include dissolved Cu ions, Cu–ligand complexes, hydrolysed/polynuclear Cu species and any ultrasmall Cu-bearing colloidal species smaller than the nominal filter cut-off. Symbols show mean values with error bars; solid lines show the best fit using an asymptotic exponential model. The figure illustrates strong matrix control over Cu mobilisation into the <0.02 μm fraction, with serum-free DMEM generating the highest measurable Cu pool.

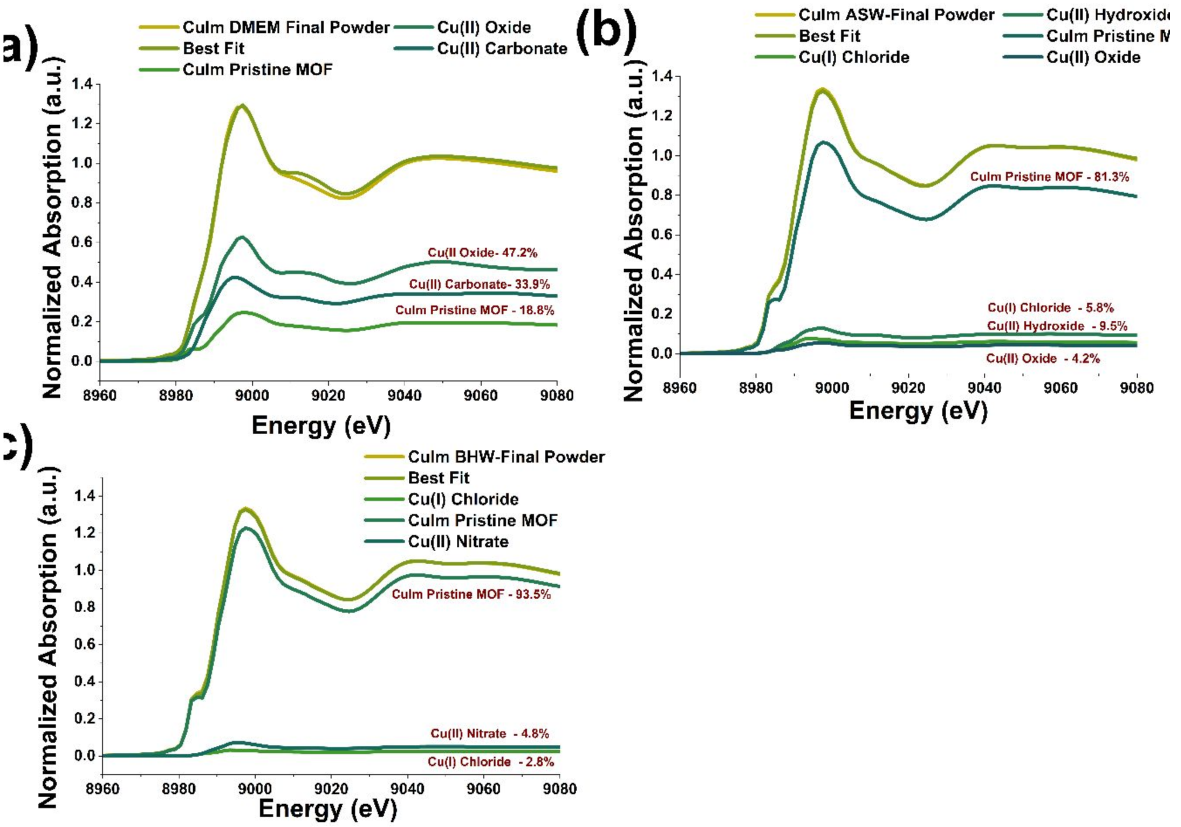

Figure S4. Linear combination fitting (LCF) of Cu speciation in 72 h endpoint ("final powder") solids across matrices. Cu K-edge XANES LCF for the **72-h end-point powders** recovered after ageing in **serum-free DMEM** (a), **ASW** (b), and **BHW** (c). Measured spectra (black) and best fits (red) are shown with contributing reference spectra and their fitted fractions (as labelled), highlighting matrix-specific terminal Cu environments reached after prolonged exposure. Corresponding LCF fit-quality parameters, including χ^2 and reduced χ^2 values for each fitted spectrum, are provided in Table S3.

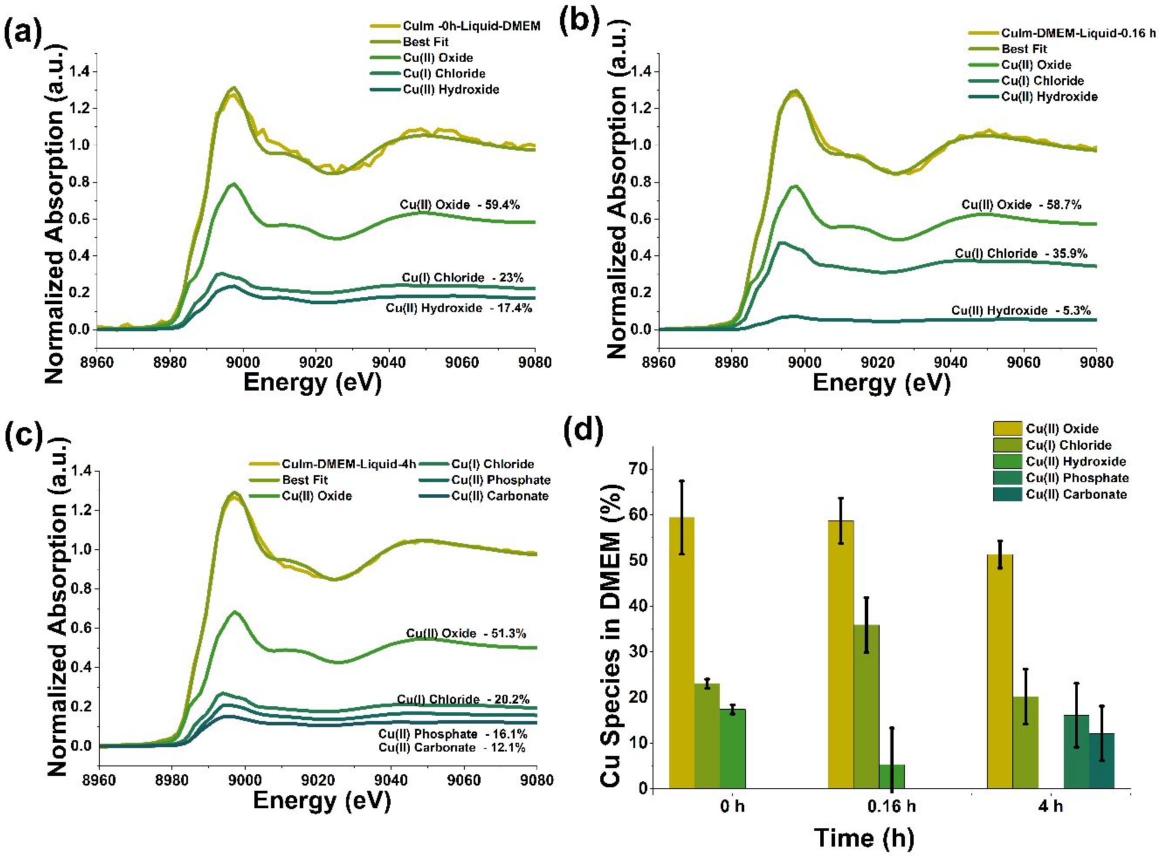

Figure S5. Time-resolved Cu speciation in the serum-free DMEM <0.02 μm filtrate by Cu K-edge XANES-LCF. Cu K-edge XANES linear combination fitting analysis for serum-free DMEM filtrates collected at representative time points: (a) 0 h, (b) 0.16 h and (c) 4 h, showing measured spectra, best fits and contributing reference components, including oxide-like, chloride-like, hydroxide-like, phosphate-associated and carbonate-associated Cu motifs. (d) Summary of LCF-derived fractions versus time, illustrating the evolution of the mobilised <0.02 μm Cu pool in the ligand-rich, protein-free matrix. The filtrate is interpreted as an operational <0.02 μm dissolved/complexed/ultrasmall-colloidal Cu fraction; therefore, these LCF components describe the average local coordination environment of Cu in this mobilised fraction and do not prove that the filtrate is purely ionic Cu or that bulk crystalline particles passed through the filter. Corresponding LCF fit-quality parameters, including χ^2 and reduced χ^2 values for each fitted spectrum, are provided in Table S3.

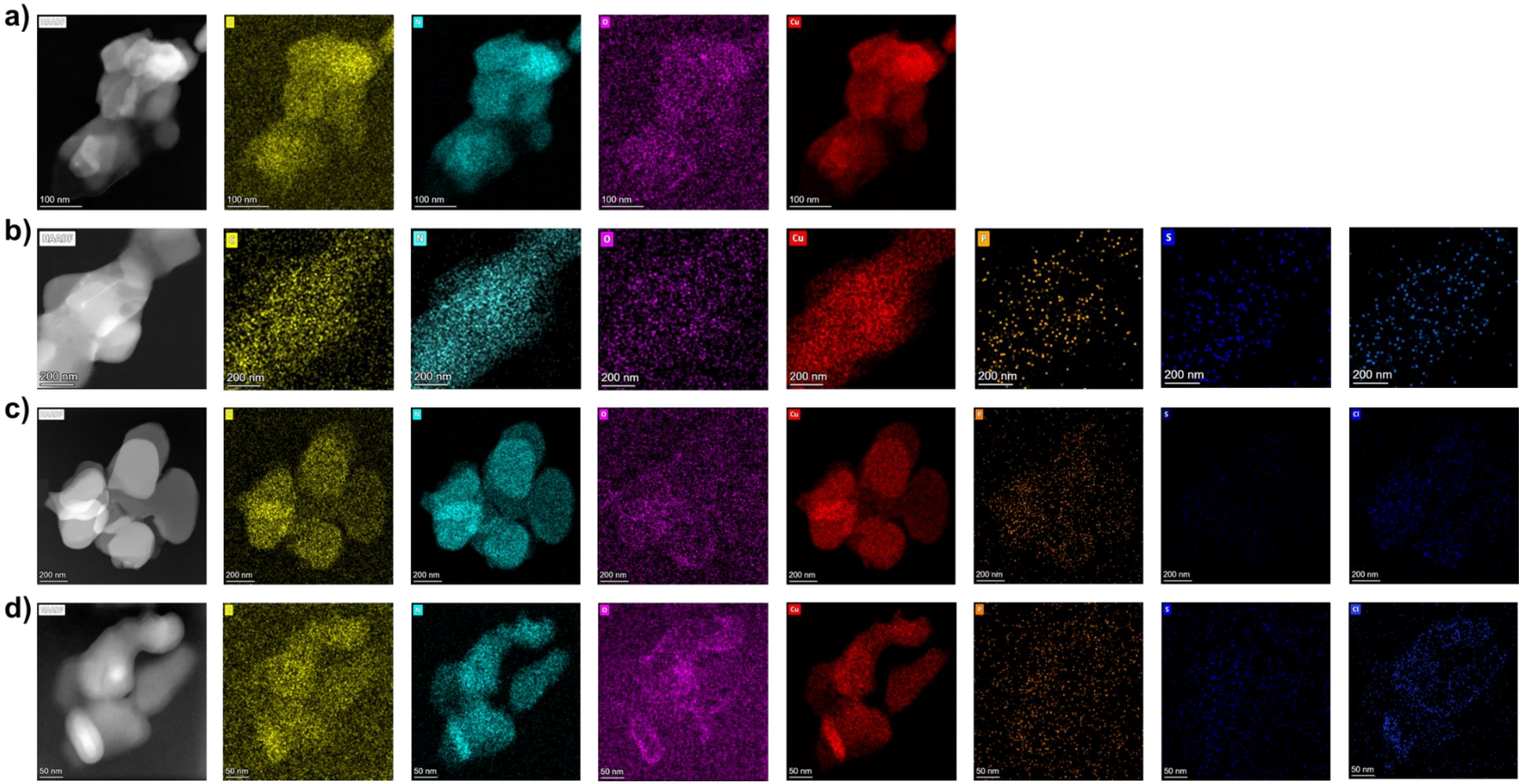

Figure S6. HAADF–STEM and STEM–EDS elemental mapping of pristine and 72 h endpoint CuIm solids**.** Representative HAADF–STEM images and corresponding STEM–EDS elemental maps for CuIm particles (pristine and/or 72 h end-point solids from the three matrices), showing spatial distributions of framework-associated elements (**C, N, O, Cu**) and selected matrix-derived elements (**P, S, Cl**, where detected). Scale bars are indicated on each panel. The maps support matrix-dependent elemental association at the particle level following ageing, complementing the bulk/surface characterisation and XAS-derived speciation trends.

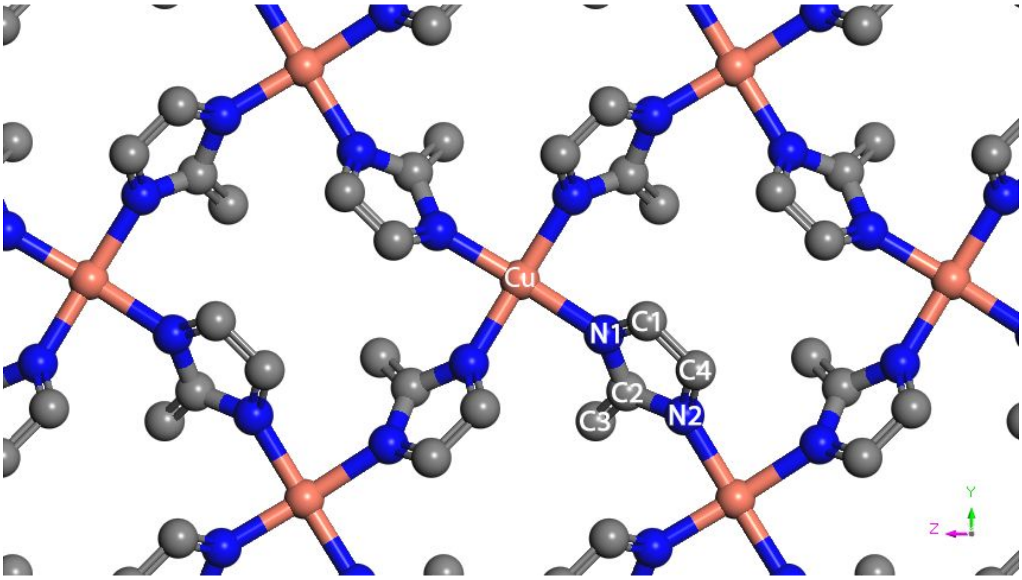

Figure S7. Schematic representation of the two-dimensional CuIm nanosheet framework. Cu centres are coordinated by imidazolate nitrogen atoms, forming the Cu-N connectivity that defines the copper-imidazolate MOF nanosheet studied in this work[1].

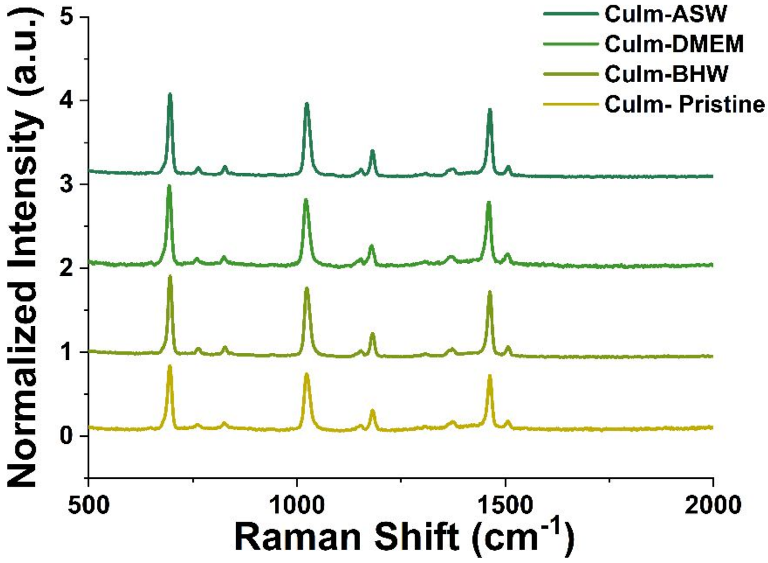

Figure S8. Raman spectra of pristine and 72 h aged CuIm solids recovered from BHW, ASW and serum-free DMEM. Raman spectra collected in the 500-2000 cm^{-1} fingerprint region show retention of the principal imidazolate-associated vibrational features after ageing in all three matrices. The persistence of bands in the ring-vibration and imidazolate fingerprint regions supports retention of the copper–imidazolate framework/linker signature after exposure, while matrix-dependent changes in relative intensity and band shape are consistent with surface conditioning and local coordination-environment reorganisation rather than complete framework decomposition.

Table S1. XPS surface atomic composition (at.%) and change relative to pristine CuIm

Δ values are absolute differences (aged – pristine CuIm).

Sample	C (at.%)	N (at.%)	O (at.%)	Cu (at.%)	ΔC	ΔN	ΔO	ΔCu
**CuIm (pristine)**	70.2	21.8	6.0	1.8	0.0	0.0	0.0	0.0
**DMEM**	63.7	14.7	19.0	2.4	−6.5	−7.1	+13.0	+0.6
**ASW**	63.4	13.4	18.8	4.2	−6.8	−8.4	+12.8	+2.4
**BHW**	65.1	22.1	9.0	3.6	−5.1	+0.3	+3.0	+1.8

Table S2. Benchmark exposure matrices used for ex-situ time-resolved CuIm transformation experiments: composition, pH, and mechanistic relevance

Matrix (abbrev.)	Key components / composition (as used here)	pH used in this study	Why this matrix (what it probes)
**Borehole water (BHW)**	Ca^{2+} (~60 mg L^{-1}), Mg^{2+} (~30 mg L^{-1}), Na^+, Cl^-, HCO_3^-, NO_3^- (<10 mg L^{-1}), trace metals (Fe, Mn), low DOC	~7.0–7.5	**Freshwater-like, carbonate/bicarbonate "hard water" baseline** to assess conservative transformation pathways and environmentally realistic conditioning under moderate ionic strength.
**Artificial seawater (ASW)**	NaCl (24.60 g L^{-1}); $NaHCO_3$ (0.180 g L^{-1}); KCl (0.670 g L^{-1}); $CaCl_2$ (1.360 g L^{-1}); $MgSO_4 \cdot 7H_2O$ (6.290 g L^{-1})	~7.5	**Marine-like, high ionic strength / major-ion matrix** to probe salt-driven surface conditioning, ion association, and medium-range reorganisation at Cu centres without strong biological ligands.
**Serum-free DMEM**	DMEM high glucose (Sigma D6429; 4.5 g L^{-1} glucose; L-glutamine; sodium pyruvate; sodium bicarbonate; phenol red) **used without FBS**	~7.2–7.4	**Physiological, ligand-rich but protein-free matrix** to decouple **ligand-driven** Cu-centre reprogramming (small-molecule ligands, bicarbonate/phosphate etc.) from protein-corona effects. (DMEM formulation per vendor; serum excluded in this study.)

Table S3. Fit-quality parameters for Cu K-edge XANES linear combination fitting (LCF) analyses shown in the main manuscript and Supporting Information. Component fractions are reported as percentages and rounded to one decimal place. χ^2 and reduced χ^2 values are reported from the least-squares LCF output. LCF was performed over the energy range 8950–9090 eV. "Particle-associated fraction" refers to the material retained on the 0.02 μm membrane filter. DMEM refers to serum-free DMEM.

Figure/panel	Sample/fraction	Time point	LCF components (%)	χ^2	Reduced χ^2
**Fig. 2a**	ASW particle-associated fraction	0 h	CuIm 79.5 ;<br>CuO-like 10.1;<br>Cu(I) chloride-like 10.3	0.01818259	1.7653×10^{-4}
**Fig. 2b**	ASW particle-associated fraction	4 h	CuIm 64.9 ;<br>CuO-like 14.0 ;<br>Cu(I) chloride-like 9.1 ;<br>Cu(II) hydroxide-like 12.1	0.01564870	1.5342×10^{-4}
**Fig. 2c**	ASW particle-associated fraction	48 h	CuIm 53.1 ;<br>CuO-like 1.8 ;<br>Cu(I) chloride-like 8.7 ;<br>Cu(II) hydroxide-like 36.4	0.02070764	2.0302×10^{-4}
**Fig. 3a**	BHW particle-associated fraction	1 h	CuIm 72.9 ;<br>Cu(I) chloride-like 14.6 ;<br>Cu(II) hydroxide-like 12.6	0.01126588	1.0938×10^{-4}
**Fig. 3b**	BHW particle-associated fraction	24 h	CuIm 67.9 ;<br>Cu(I) chloride-like 6.5 ;<br>Cu(II) hydroxide-like 25.7	0.01927467	1.8713×10^{-4}
**Fig. 3c**	BHW particle-associated fraction	72 h	CuIm 67.9 ;<br>Cu(I) chloride-like 11.2 ;<br>Cu(II) hydroxide-like 20.9	0.01668387	1.6198×10^{-4}
**Fig. 4a**	DMEM particle-associated fraction	0 h	CuIm 87.4 ;<br>Cu(I) chloride-like 12.6	0.05805939	5.5826×10^{-4}
**Fig. 4b**	DMEM particle-associated fraction	0.5 h	CuIm 76.9 ;<br>Cu(I) chloride-like 11.7 ;<br>Cu(II) carbonate-like 11.4	0.03056114	2.9671×10^{-4}
**Fig. 4c**	DMEM particle-associated fraction	4 h	CuIm 28.6 ;<br>CuO-like 33.5 ;	0.00784935	7.6954×10^{-5}

			Cu(I) chloride-like 5.5 ;<br>Cu(II) hydroxide-like 32.4		
**Fig. 4d**	DMEM particle-associated fraction	48 h	CuIm 23.1 ;<br>CuO-like 34.9 ;<br>Cu(II) carbonate-like 14.6 ;<br>Cu(II) hydroxide-like 27.5	0.00613867	6.0183×10^{-5}
**Fig. S4a**	DMEM final powder	72 h endpoint	CuIm 18.8 ;<br>CuO-like 47.2 ;<br>Cu(II) carbonate-like 33.9	0.02510428	2.4373×10^{-4}
**Fig. S4b**	ASW final powder	72 h endpoint	CuIm 81.4 ;<br>CuO-like 4.2 ;<br>Cu(I) chloride-like 5.9 ;<br>Cu(II) hydroxide-like 9.6	0.00285840	2.8301×10^{-5}
**Fig. S4c**	BHW final powder	72 h endpoint	CuIm 93.6 ;<br>Cu(I) chloride-like 2.4 ;<br>Cu(II) nitrate-like 4.8	0.00135546	1.3289×10^{-5}
**Fig. S5a**	DMEM filtrate, <0.02 µm fraction	0 h	CuO-like 59.4 ;<br>Cu(I) chloride-like 23.1 ;<br>Cu(II) hydroxide-like 17.5	0.07387161	7.7760×10^{-4}
**Fig. S5b**	DMEM filtrate, <0.02 µm fraction	0.16 h	CuO-like 58.7 ;<br>Cu(I) chloride-like 35.9 ;<br>Cu(II) hydroxide-like 5.4	0.02396750	2.5497×10^{-4}
**Fig. S5c**	DMEM filtrate, <0.02 µm fraction	4 h	CuO-like 51.3; Cu(I) chloride-like 20.3; Cu(II) phosphate-like 16.2; Cu(II) carbonate-like 12.2	0.01595901	1.6978×10^{-4}

References

(1) Bhadane, P.; Menon, D.; Goyal, P.; Reza Alizadeh Kiapi, M.; Kanta Satpathy, B.; Lanza, A.; Mikulska, I.; Scatena, R.; Michalik, S.; Mahato, P.; Asgari, M.; Chen, X.; Chakraborty, S.; Mishra, A.; Lynch, I.; Fairen-Jimenez, D.; Misra, S. K. A Two-Dimensional Metal-Organic Framework for Efficient Recovery of Heavy and Light Rare Earth Elements from Electronic Wastes. *Sep. Purif. Technol.* 2025, *360*, 130946. https://doi.org/10.1016/J.SEPPUR.2024.130946.
